# Parent‐led CBT delivered via online and telephone support alongside usual school practice versus usual school practice only for young children identified as at risk for anxiety disorders through screening in schools: a cluster randomised controlled trial

**DOI:** 10.1111/jcpp.70119

**Published:** 2026-02-19

**Authors:** Tessa Reardon, Obioha C. Ukoumunne, Helen Dodd, Gemma Halliday, Claire Hill, Bec Jasper, Benjamin Jones, Peter J. Lawrence, Fran Morgan, Anna Placzek, Ronald M. Rapee, Mara Violato, Shuye Yu, MYCATS Team, Emily Davey, Jeni Fisk, Iheoma Green, Laura Hankey, Elizabeth Hindhaugh, Chloe Hooper, Lindsey Martineau, Amy McCall, Natascha Niekamp, Natasha Pall, Samantha Pearcey, Ruth Potts, Abigail Thomson, Tamatha Weisser, Joshua Wright, Siyu Zhou, Cathy Creswell

**Affiliations:** ^1^ Department of Experimental Psychology University of Oxford Oxford UK; ^2^ Department of Psychiatry University of Oxford Oxford UK; ^3^ NIHR ARC South West Peninsula (PenARC) University of Exeter Exeter UK; ^4^ Exeter Medical School University of Exeter Exeter UK; ^5^ School of Psychology & Clinical Language Sciences University of Reading Reading UK; ^6^ Parents and Carers Together Suffolk UK; ^7^ Centre for Innovation in Mental Health, School of Psychology University of Southampton Southampton UK; ^8^ Square Peg East Sussex UK; ^9^ Lifespan Health and Wellbeing Research Centre, School of Psychological Sciences Macquarie University Sydney NSW Australia; ^10^ Health Economics Research Centre, Nuffield Department of Population Health University of Oxford Oxford UK; ^11^ Centre for Health Economics University of York York UK

**Keywords:** Anxiety, early intervention, prevention, screening, online intervention, parent‐led intervention

## Abstract

**Background:**

Providing accessible CBT for young children identified as at risk for anxiety disorders through screening in schools could reduce later problems. This study aimed to evaluate the effectiveness of parent‐led CBT delivered via online and telephone call support alongside usual school provision, compared to usual school provision only for young children identified through screening as having at least one risk.

**Methods:**

We conducted a pragmatic, parallel group, superiority cluster randomised controlled trial in 95 primary/infant schools in England. Parents of children (aged 4–7) in sampled classes completed screening, and children who screened positive for one or more risks (anxiety symptoms/inhibition/parent anxiety) were eligible for the trial. Schools (clusters) were randomised (1:1) to intervention or usual school practice, stratified by school‐level deprivation. Schools in both arms continued with usual provision, and parents in intervention schools were offered parent‐led CBT via online and telephone support. The primary outcome was the presence of an anxiety disorder diagnosis at 12 months, assessed via the ADIS‐P administered by independent assessors. Secondary clinical outcomes included parent‐reported child anxiety symptoms, related interference, externalising symptoms, additional risks and intervention targets at 12 weeks and 12 months. Primary analyses were conducted on the full intention‐to‐treat population. The trial was prospectively registered with ISRCTN 82398107.

**Results:**

In total, 2,328 children were screened; 1,172 were eligible; 865 enrolled. Forty‐eight schools (434 children) were assigned to intervention and 47 schools (431 children) to usual school practice. At 12 months, the overall frequency of anxiety disorders was low, 6.8% (21/310) of children in the intervention arm compared to 11.5% (36/312) in the usual school practice arm; this difference was not statistically significant (adjusted odds ratio 0.67 [0.37 to 1.21], *p* = .19). However, the intervention was superior to usual school practice across all secondary outcomes (standardised mean difference: 0.15 to 0.47 at 12 weeks; 0.19 to 0.41 at 12 months). No serious adverse events were reported.

**Conclusions:**

Although the intervention did not significantly reduce anxiety disorders at 12 months, improvements across all other assessed outcomes indicate this approach brings wider immediate benefits and reduces known risks for future anxiety disorders. Future research needs to consider longer‐term preventative effects.

## Introduction

Anxiety problems in children have adverse consequences for future mental and physical health, education and employment outcomes, and associated societal costs (Pollard et al., 2023). Timely intervention at or before the first onset of mental health disorders can reduce associated negative consequences for individuals and society (Fusar‐Poli et al., 2021). With 38% of anxiety disorders starting before age 14 and the peak age of onset of 5.5 years (Solmi et al., 2022), early intervention and prevention for anxiety disorders need to target young children. Cognitive behavioural therapy (CBT) interventions are effective for preventing and treating anxiety disorders in children as young as 3–4 years (Howes Vallis, Zwicker, Uher, & Pavlova, 2020; James, Reardon, Soler, James, & Creswell, 2020). However, the considerable barriers to identifying and seeking support for early signs of anxiety problems in children (Lawrence, Harvey, Williams, & Creswell, 2022; Reardon, Harvey, & Creswell, 2020) and oversubscribed services mean few young children receive these interventions (Children's Commissioner, 2024; Reardon et al., 2020). Maximising both reach and efficiency are therefore essential for early intervention and prevention programmes.

Delivering interventions through schools removes key barriers to seeking and accessing support from external mental health services. Universal school‐based interventions reach whole classes, but there is only weak evidence of positive effects on anxiety in primary schools (Caldwell et al., 2019) and any reductions in average self‐reported anxiety symptoms may not apply for children with elevated anxiety (Stallard et al., 2014). Anxiety interventions for young children under 8 years tend to be delivered via parents (Howes Vallis et al., 2020; James et al., 2020) which has the advantage of efficient delivery (Creswell et al., 2017, 2024), but cannot easily be provided in whole‐class settings. An alternative to universal interventions is to offer screening universally through schools, which minimises identification and help‐seeking barriers, and then provide targeted parent‐led interventions for children identified as at heightened risk for anxiety disorders.

Elevated anxiety symptoms, inhibited temperament and parental anxiety disorders are the most well established individual risks for future anxiety disorders (Lawrence, Murayama, & Creswell, 2019; Sandstrom, Uher, & Pavlova, 2020; Steinsbekk, Ranum, & Wichstrøm, 2022). There is some evidence to support interventions for primary school‐aged children in the context of each these risks (Ginsburg, Drake, Tein, Teetsel, & Riddle, 2015; Mifsud & Rapee, 2005; Rapee, Kennedy, Ingram, Edwards, & Sweeney, 2005). However, no study has screened for all three of these risks and provided an intervention in the context of any one or combination of risks, which would maximise reach. Existing trials that have evaluated parent‐only CBT interventions for young children under 8 years identified as at risk for anxiety disorders have primarily focused on inhibited temperament, selecting children on the basis of this single risk (Bayer et al., 2018; Chronis‐Tuscano et al., 2022; Morgan et al., 2017; Rapee et al., 2005), with one trial also requiring the presence of a parent anxiety disorder (Kennedy, Rapee, & Edwards, 2009). Most trials have recruited families through advertisements which raises questions about reach, or incorporated laboratory assessments to identify eligible children that would not be feasible to implement in routine practice. The one trial that conducted universal screening for inhibition via preschools (Bayer et al., 2018) encouragingly found that offering CBT via group sessions for parents of behaviourally inhibited children reduced parent‐reported child anxiety symptoms over 2 years compared to usual care (Bayer et al., 2021). The frequency of anxiety disorders and parent‐reported measures of targeted parent behaviours did not significantly change at 1‐ or 2‐year follow‐up (Bayer et al., 2018, 2021). Attendance at parent sessions in this study was relatively low (only 34% attended most sessions).

Delivering interventions for parents remotely and via digital platforms has the potential to address logistical and stigma‐related barriers associated with in‐person and group sessions, as well as improving efficiency (Creswell et al., 2024). One trial has evaluated an online CBT intervention for parents of behaviourally inhibited children under 8 years and found promising reductions in anxiety disorders and parent‐reported child anxiety symptoms over 6 months (Morgan et al., 2017). Notably, this intervention was largely self‐guided, with few parents (5%) requesting the optional additional telephone support, and completion of online modules was relatively low (25% completed all online content). Providing therapist support alongside self‐help resources has been shown to improve retention and treatment outcomes compared to self‐help resources alone for children with anxiety disorders (Rapee, Abbott, & Lyneham, 2006) so the same may apply in the context of at‐risk populations. No previous trials have evaluated outcomes from providing therapist‐supported online CBT for parents of young children identified as at risk for anxiety disorders.

The primary aim of this study was to determine the effectiveness of providing parent‐led CBT via online and telephone support alongside usual school provision (intervention) compared to usual school provision only (usual school practice) for parents of young children (aged 4–7 years) identified as at risk for anxiety disorders on the basis of at least one risk (elevated child anxiety, and/or inhibition, and/or parent anxiety) through a universal offer of screening in schools. We hypothesised that there would be fewer children with anxiety disorders in the intervention arm compared to the usual school practice arm at 12 months. We also set out to investigate whether the intervention brings wider benefits for at‐risk children compared to usual school practice. Secondary objectives included establishing effects for: (a) risks for the development of future anxiety disorders (child anxiety symptoms, inhibition, parent anxiety symptoms); (b) broader child clinical outcomes, including interference related to child anxiety (as this is a strong predictor of anxiety disorder status among primary school children; Reardon et al., 2025) and a priority for families (Creswell et al., 2021) and externalising symptoms which have been shown to reduce following other forms of anxiety‐focused CBT (Kreuze, Pijnenborg, De Jonge, & Nauta, 2018); and (c) additional parent and child behaviours that the intervention targets, including parent overprotection and parenting self‐efficacy, and child behavioural avoidance, intolerance of uncertainty and coping efficacy. In line with previous trials in this age group and because of a lack of validated self‐report measures for children <8 years, secondary outcomes were assessed using parent‐report questionnaires. To allow detailed reporting, full economic analysis, qualitative data and analyses examining moderators and mediators of outcomes will be reported elsewhere.

## Methods

### Study design and participants

We conducted a pragmatic, parallel arm, superiority cluster randomised controlled trial in 95 mainstream primary/infant schools in England, with schools (clusters) randomised to intervention and usual school practice arms. Cluster randomisation was used to prevent contamination between families and to enhance acceptability within schools. To be eligible, schools needed to have at least two classes per target year group (Reception, Year 1 and Year 2; children aged 4–7 years).

All parents of children in sampled classes were invited to complete screening measures to assess three risks (see below) and to provide demographic information about the child and parent. Children who screened positive on at least one risk were eligible for the trial, with a maximum of one child per family because the intervention is parent‐led and outcomes were parent‐reported. The only additional requirements were that parents needed sufficient English to provide consent, complete measures and participate in the intervention, and they needed frequent access to the internet; we sought to find potential solutions where these issues arose.

Parents of eligible children were invited to take part in the trial and those who provided written consent and completed baseline questionnaires were enrolled. Recruitment was staggered for pragmatic reasons, with schools and participants recruited in five separate recruitment cohorts to ensure sufficient capacity within the team to support subsequent data collection and intervention delivery activities.

Ethical approval was obtained from the University of Oxford Medical Sciences Interdivisional Research Ethics Committee (Reference: R62531). The study was prospectively registered on ISRCTN (82398107), and the protocol (Reardon et al., 2022) and statistical analysis plan (Jones et al., 2022) were published.

### Screening

Child anxiety symptoms were assessed using the 28‐item Preschool Anxiety Scale (PAS) (Spence, Rapee, McDonald, & Ingram, 2001) and children who scored ≥34 (out of a possible 112) screened positive for child anxiety symptoms. This cut‐off represents a T‐score of 60, derived from normative data for children aged 3 to 6.5 years to indicate elevated child anxiety (see https://www.scaswebsite.com for further details). Behavioural inhibition was assessed using the 7‐item Approach subscale of the Short Temperament Scale for Children (STSC‐A) (Sanson, Pedlow, Cann, Prior, & Oberklaid, 1996). In line with the previous prevention trial that screened for inhibition in preschool settings (Bayer et al., 2018), scores ≥30 out of a possible 42 on the STSC‐A were classified as screening positive for behavioural inhibition. The 7‐item Generalised Anxiety Disorder Scale (GAD‐7) was used to assess parent anxiety, with scores ≥8 (out of a maximum possible score of 21) classified as screening positive for parent anxiety on the basis that this is the optimal cut‐off for identifying cases of any type of anxiety disorder in adults (Kroenke, Spitzer, Williams, Monahan, & Löwe, 2007; Plummer, Manea, Trepel, & McMillan, 2016).

Where two or more children in a family or household screened positive on one or more screening questionnaire, the child with the highest score on the PAS was eligible for the trial; if these children had equal PAS scores, the child with the highest STSC‐A score was eligible.

### Class sampling, randomisation and masking

Class sampling and school randomisation were conducted by an independent statistician otherwise unconnected to the trial, using computer‐based algorithms written using R software.

Where schools had two classes per eligible year group, all six classes participated in screening. In larger schools, two classes per eligible year group were randomly sampled.

School randomisation was conducted after screening and baseline assessments were completed in a cohort of schools. Schools were randomly allocated to the two arms in a 1:1 ratio, stratified according to school‐level deprivation (above/below national median of 15.8% for percentage of pupils eligible for free school meals, in 2019 (UK Government, 2019)) using block randomisation with block sizes of two and four. To minimise imbalance in the number of participants across arms, schools were ordered according to the number of participants before randomisation. The independent statistician shared randomisation outcomes with the study team who then informed schools and families.

It was not possible for schools, participants, clinicians or members of the study team who were in contact with schools and families throughout to be blind to trial arm. Diagnostic assessments at 12 months were administered and supervised by independent assessors who were blind to group allocation and participants were asked not to reveal their allocation during the interview. Where participants did reveal their arm (13%, 81/622), the supervisor remained blind until diagnostic outcomes were agreed. Trial statisticians remained blind to group allocation up until final datasets were locked for analysis.

### Procedures

We obtained written agreement for the school's involvement from the headteacher and each school nominated at least one ‘MYCATS Lead’ as the main point of contact for the study team. Schools distributed electronic study information in written and video formats to all parents of children in participating classes. The study team worked with schools to identify additional strategies to promote participation in screening (e.g. displaying posters with a QR code at school, offering online information sessions for parents, sending reminders). Parents who provided consent for screening and their contact details via an online e‐consent framework were sent online screening questionnaires and a form to provide demographic information (including child and parent gender: female/male/other/prefer not to say, and child and parent ethnicity). Parents received electronic written feedback informing them whether their child was eligible, and the study team arranged a telephone call with parents of eligible children. Parents who verbally agreed were asked to provide written online consent for the trial. After trial consent was obtained, parents were sent a link to baseline questionnaires. Parent‐report screening and baseline questionnaires were collected using a secure web‐based system (Harris et al., 2019). Schools nominated a member of staff to complete a bespoke questionnaire to provide information about the school's social, emotional, mental health and well‐being provision (Appendices S1 and S2). Schools received £3 per enrolled family as a thank you for support with recruitment.

A full schedule of enrolment, interventions and assessment is provided in the protocol (Reardon et al., 2022). Parents completed follow‐up online questionnaires at 6‐week, 12‐week and 12‐month post‐randomisation, using the same secure web‐based system as screening and baseline. At 12 months, parents were also asked to complete a diagnostic assessment for their child over the telephone or video call. Regular newsletters were sent to schools and families, and we used reminder emails, text messages and telephone calls to promote completion of follow‐ups. After each follow‐up, parents received a £10 voucher to say thank you.

Parents in schools allocated to the intervention arm were offered a therapist‐supported, parent‐led CBT programme that was delivered via online modules and telephone/video calls (OSI: Online Support and Intervention for child anxiety). The intervention content is based on a parent‐led CBT face‐to‐face treatment for child anxiety disorders (Creswell et al., 2017), and the online version was developed using a user‐centred design approach (Hill, Reardon, Taylor, & Creswell, 2022). Minor adaptations were made to the online content specifically for young children at risk for anxiety disorders (Appendix S3). The intervention focuses on supporting parents to use cognitive behavioural strategies to help their child to understand fears and worries, test out anxious thoughts in a supportive and gradual way, and problem solve. Parents work through seven online modules, and each online module is supported by a short weekly call and a follow‐up call about 4 weeks after the final content. Online modules present information in varied formats, including simple written text, videos, animations, audio recordings of the text, interactive activities and include built‐in outcome measures. Parents also have access to an optional mobile game app for children which they can use to help motivate their child to participate in intervention activities. Therapists can view and monitor parents' responses to activities and questionnaires.

Trial therapists were five qualified Children's Wellbeing Practitioners (CWPs). CWPs (NHS, band 5) are trained to provide low‐intensity psychological therapies for children, including parent‐led CBT for child anxiety problems, through a one‐year graduate training programme. For the trial, therapists received specific training in OSI through a written manual with highly structured and standardised guidance, discussion and role‐play. Therapists attended weekly supervision with a clinical psychologist. Supervisors closely monitored adherence throughout, including through the use of audio‐recorded support calls in supervision where parents consented to this. CWPs and supervisors completed logs to record time spent on intervention activities.

Across both arms, families were free to continue to seek and access any support or care as usual and schools continued to provide any usual support and interventions. After the 12‐month assessment, families in the usual school practice arm were provided with text and audio versions of the OSI content.

### Outcomes

The primary outcome was the presence/absence of an anxiety disorder diagnosis at 12 months, on the basis of the Anxiety Disorders Interview Schedule for children‐Parent Interview (ADIS‐P) (Silverman & Albano, 1996). The ADIS is widely used in trials evaluating CBT for child anxiety disorders (James et al., 2020), and the parent interview conducted via telephone can reliably establish the presence/absence of anxiety disorders in young children (Lyneham & Rapee, 2005). Minor adaptations were made to the interview to be consistent with DSM‐5 criteria for anxiety disorder diagnoses. A team of trained assessors blind to trial arm conducted interviews. Final diagnoses and Clinical Severity Ratings (CSRs) (0–8) for all interviews were assigned following team discussion led by an experienced diagnostician who was also blind to trial arm status. In line with standard guidance, ‘anxiety diagnosis’ was recorded if a child was assigned an anxiety disorder diagnosis with CSR 4–8 and ‘no anxiety diagnosis’ if a child was not assigned any anxiety disorder diagnosis with CSR 4–8. All diagnostic interviews were doubled‐rated by the assessor and experienced diagnostician, and inter‐rater reliability within the assessment team was high (Kappa = .85 for diagnosis, and intra‐class correlation coefficient (ICC) for CSRs = 0.91).

Secondary clinical outcomes were assessed using parent‐reported questionnaire measures at 12 weeks and 12 months, including the following: (a) risk factors—child anxiety symptoms (PAS, Spence et al., 2001), behavioural inhibition (STSC‐A, Sanson et al., 1996), parent anxiety (GAD‐7, Spitzer, Kroenke, Williams, & Löwe, 2006); (b) broader clinical outcomes—interference related to the child's anxiety (Child Anxiety Life Interference Scale‐preschool version; CALIS‐PV, Gilbertson, Morgan, Rapee, Lyneham, & Bayer, 2017), externalising symptoms (Strengths and Difficulties Questionnaire Externalising Scale; SDQ‐E, Goodman & Goodman, 2009); (c) additional intervention targets—parental overprotection (Parent Overprotection Measure; POM, Rapee, Edwards, Mabood, & Freeman, 2024), parent self‐efficacy (self‐efficacy subscale of the Parenting Sense of Competence Scale (PSOC‐SE, Johnston & Mash, 1989), child behavioural avoidance (Child Avoidance Measure; CAMP, Whiteside, Gryczkowski, Ale, Brown‐Jacobsen, & McCarthy, 2013), child intolerance of uncertainty (Responses to Uncertainty and Low Environmental Structure; RULES, Sanchez et al., 2017) and child coping efficacy (Coping Questionnaire; CQ, Crane & Kendall, 2020)) (see Appendix S4 for further details on secondary clinical outcome measures).

Parents' experiences of the intervention and trial procedures were assessed using a bespoke parent‐reported acceptability questionnaire at baseline, 12 weeks and 12 months. Acceptability questionnaires asked participants to rate the extent to which they agreed with a series of statements and also provide written feedback in free text boxes.

Adverse events were monitored and recorded by the study team throughout. Parents were also asked specific questions about possible harms related to the study and (where applicable) the intervention on the 12‐week and 12‐month acceptability questionnaire.

### Statistical analysis

We originally aimed to recruit 1,080 children from 60 schools (30 schools and 540 children per arm). This is a large enough sample to detect a reduction in the presence of anxiety disorders from 50% in the usual school practice arm to 35% in the intervention arm, with 90% power at the 5% (2‐sided) level of significance. This difference would be meaningful to detect and was consistent with the previous anxiety prevention trial for young children that incorporated universal screening in preschools which reported 50% prevalence rate for anxiety disorders at 1‐year follow‐up among children previously identified as ‘inhibited’ (Bayer et al., 2018). The sample size calculation assumed 80% of trial participants would complete the 12‐month diagnostic assessment and allowed for clustering in schools. We assumed an intra‐cluster (intra‐school) correlation coefficient of 0.05 which is a conservative estimate compared to reports of the intra‐school correlation coefficient for pupil mental health and well‐being outcomes (Parker et al., 2025).

This target sample size was also based on assumptions related to recruitment within schools so we planned to review these assumptions following initial recruitment and increase the number of schools if needed (Reardon et al., 2022). After reviewing the recruitment rate in the first two cohorts, we expected to need to include 86 schools to allow us to recruit 876 trial participants and provide approximately 88% power at the 5% (2‐sided) level of significance to detect a reduction in the presence of anxiety disorders at 12 months from 50 to 35% (Jones et al., 2022).

Analyses were undertaken as detailed in the pre‐specified analysis plan (Jones et al., 2022).

Characteristics of schools and participants are reported using means and standard deviations or medians and ranges for continuous variables and frequencies and percentages for categorical variables.

In the main analysis, the primary and secondary outcomes were compared between the intervention and usual school practice arms, using the intention‐to‐treat principle with participants analysed based on the trial arm that their cluster was allocated.

The main analyses of the primary and secondary outcomes were based on analyses of multiple imputed data sets. Fifty data sets were imputed using the *pan* package (Grund, Ludtke, & Robitzsch, 2016) in *R* software (R Core Team, 2020), by fitting a multivariate linear mixed effects (‘multilevel’) model that recognises the correlation between the responses of participants from the same cluster. The imputation model included all outcomes at all study waves, prognostic factors that were adjusted for, trial arm status, and the number of OSI modules completed (auxiliary variable). The imputed data were analysed in *Stata* software (StatCorp, 2022) using the *mi* suite of commands.

Sensitivity analyses including only participants that provided data (complete case analyses) were also undertaken for all outcomes. For the primary outcome only, a sensitivity analysis was undertaken to estimate the complier average causal effect (CACE) in the population of participants that comply with the intervention with compliance defined as completion of at least the first five online modules (Modules 0 to 4). A further sensitivity analysis was undertaken for the primary outcome, restricting the analysis to participants that provided data within the 12‐week data collection window. The CACE analysis and the restricted analysis were undertaken both using imputed data and complete cases.

The primary outcome (binary) was compared between trial arms using marginal models using Generalised Estimating Equations (GEEs) with information sandwich (‘robust’) standard errors. Odds ratios were reported for the estimated effect. The secondary outcomes (all continuous) were compared using linear mixed effects models estimated using restricted maximum likelihood. Mean differences and standardised mean differences were reported for the estimated effect. The GEEs and mixed model methods allow for correlation between the responses of participants from the same cluster. Unadjusted (crude) analyses and analyses that were adjusted for potential prognostic factors (free school meal status of the school, number of participants in the school, cohort, year group, child gender, decile of the index of multiple deprivation rank based on the family's address and, where available, the baseline response for the outcome measure) were undertaken. The adjusted analyses are the main ones. Intra‐cluster (intra‐school) correlation coefficients (ICCs) are reported for the unadjusted and adjusted analyses based on the complete case analysis results.

For the CACE analysis, a two‐stage least squares instrumental variable approach was used to estimate the complier average causal effect, using the bootstrap method with 400 replications to obtain bias corrected confidence intervals.

Acceptability questionnaire responses were summarised by trial arm and overall using numbers and percentages. Free text feedback provided on acceptability questionnaires was coded according to the explicit content described and grouped into categories to enable frequency counts.

## Results

**Figure 1:**
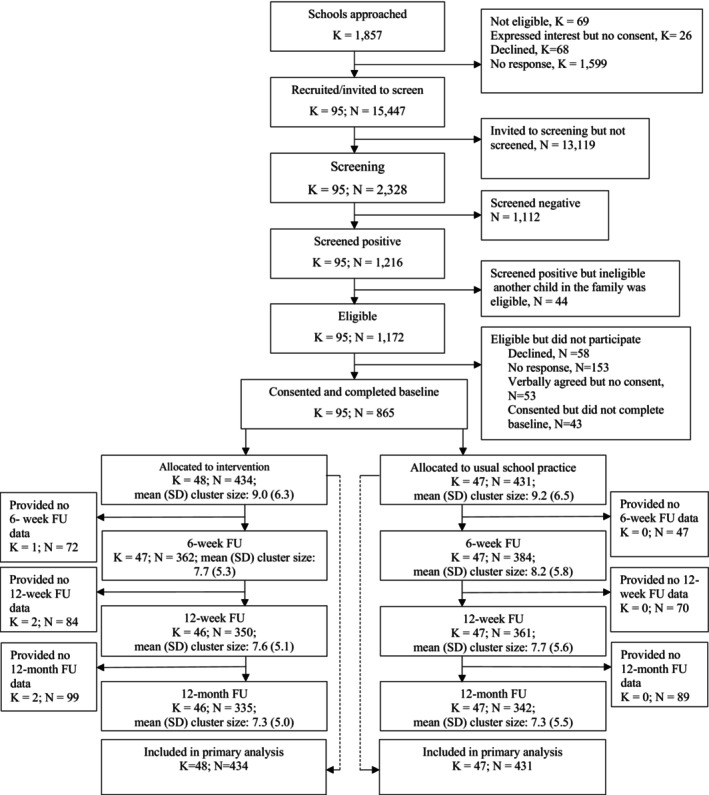
Trial profile. *K* = number of schools. *N* = number of children. FU = follow‐up. Where participants did not complete a follow‐up, they were invited to complete subsequent follow‐ups unless they withdrew from the trial. 13 intervention arm participants and 4 usual school practice participant withdrew before completing the 6‐week follow‐up; 0 intervention arm participants and 2 usual school practice participants withdrew after the 6‐week follow‐up and before completing the 12‐week follow‐up

We obtained agreement from 95 schools between 8 February 2021 and 29 June 2022 (see Figure 1). Participants were screened from 21 March 2021 to 21 July 2022, and baseline assessments were collected from 14 April 2021 to 28 July 2022.

Parents of 2,328 children (15% of invited) completed screening questionnaires, 1,172 children (50% of screened) were eligible and parents of 865 (74% of eligible) consented and completed baseline questionnaires (see Tables S1 and S2 for characteristics of those who did and did not enrol). Forty‐eight schools (434 children) were randomised to the intervention arm and 47 schools (431 children) were randomised to the usual school practice arm. Parents of 332 (76%) children in the intervention arm completed the core intervention content (Table S3) and the mean amount of therapist time to deliver support calls was 152 min per family (Table S4). The primary outcome (diagnostic assessment) was completed at 12 months for 310 (71%) children in 46 (96%) schools in the intervention arm and 312 (72%) children in 47 (100%) schools in the usual school practice arm (see Table S5 for completion rates for secondary outcomes). Inspection of baseline characteristics for children with and without 12‐month diagnostic outcome data indicates those with lower functioning on clinical measures and from more deprived backgrounds and single parent households were over‐represented among children without diagnostic assessment data (Tables S6 and S7).

Baseline characteristics for schools and participants are provided in Tables 1 and 2. Schools were from across England and had a varied demographic profile, with 45 (47%) and 29 (31%) above the national median proportion of pupils eligible for free school meals (proxy indicator of deprivation) and pupils with English as an additional language, respectively. School‐reported social, emotional, mental health and well‐being provision was similar across arms. Children had a mean (*SD*) age of 6.2 (0.9) years, with 254 (29%) in Reception (age 4–5 years), 297 (34%) in Year 1 (5–6 years) and 314 (36%) in Year 2 (6–7 years). There were almost equal numbers of female children (*n* = 432) and male children (*n* = 433), and mostly female parents (810, 94% female). In total, 751 (87%) children and 775 (90%) parents were described as White, compared to 82% of people in England and Wales (UK Government, 2022). There was variation on indicators of socioeconomic background, although families living in the most deprived areas and on low incomes were slightly underrepresented (145 [17%]) lived in the 20% most deprived areas; 311 out of 760 [41%] had a household income ≤£40,000 where the national median household income was approximately £35,000 (Office for National Statistics, 2023).

**Table 1:** Baseline characteristics of schools

	Intervention (*N* = 48)	Usual school practice (*N* = 47)	Total (*N* = 95)
Cohort			
Cohort 1	6 (13%)	7 (15%)	13 (14%)
Cohort 2	12 (25%)	10 (21%)	22 (23%)
Cohort 3	12 (25%)	13 (28%)	25 (26%)
Cohort 4	13 (27%)	14 (30%)	27 (28%)
Cohort 5	5 (10%)	3 (6%)	8 (8%)
Region in England			
North‐West/North‐East	11 (23%)	9 (19%)	20 (21%)
West‐Midlands/East‐Midlands	16 (33%)	14 (30%)	30 (32%)
Eastern	6 (13%)	5 (11%)	11 (12%)
South‐West	6 (13%)	5 (11%)	11 (12%)
Greater London and South East	7 (15%)	11 (23%)	18 (19%)
Yorkshire and Humber	2 (4%)	3 (6%)	5 (5%)
Size			
Number of pupils on school roll, mean (*SD*)	421 (154)	462 (147)	441 (151)
Number of eligible classes, median (range)	6 (6 to 12)	6 (6 to 15)	6 (6 to 15)
Deprivation			
Above national median of pupils eligible for free school meals^a^	23 (48%)	22 (47%)	45 (47%)
Percentage of pupils eligible for free school meals, mean (*SD*)	17.4 (11.8)	17.9 (10.3)	17.6 (11.0)
Language			
Above national median of pupils with English as an additional language^b^	13 (27%)	16 (34%)	29 (31%)
Percentage of pupils with English as an additional language, mean (*SD*)	17.7 (20.3)	22.1 (23.4)	19.9 (21.9)
Special educational needs			
Percentage of pupils with Special Educational Needs Support, mean (*SD*)	13.7 (7.8)	14.2 (6.1)	13.9 (7.0)
Percentage of pupils with an Education Health Care Plan, mean (*SD*)	1.2 (0.9)	1.3 (0.9)	1.2 (0.9)
Social, emotional and mental health and wellbeing provision, mean (*SD*)^c^	8.27 (3.9)	8.23 (4.3)	8.25 (4.1)

Data are *n* (%) unless otherwise stated.

^a^ National median for primary schools in England in 2019 was 15.8%.

^b^ National median for primary schools in England in 2019 was 21.2%.

^c^ Data available for 44 out of 48 intervention arm schools and 40 out of 47 usual school practice schools. Total possible score 0 to 30 (see Appendices S1 and S2).

**Table 2:** Baseline characteristics of participants

	Intervention (*N* = 434)	Usual school practice (*N* = 431)	Total (*N* = 865)
Cohort			
Cohort 1	51 (12%)	58 (13%)	109 (13%)
Cohort 2	126 (29%)	125 (29%)	251 (29%)
Cohort 3	118 (27%)	118 (27%)	236 (27%)
Cohort 4	99 (23%)	118 (27%)	217 (25%)
Cohort 5	40 (9%)	12 (3%)	52 (6%)
Child year group^a^			
Reception, 4–5 years	124 (29%)	130 (30%)	254 (29%)
Year 1, 5–6 years	152 (35%)	145 (34%)	297 (34%)
Year 2, 6–7 years	158 (36%)	156 (36%)	314 (36%)
Child age, mean (*SD*) years^b^	6.3 (0.9)	6.2 (0.9)	6.2 (0.9)
Child gender			
Female	219 (50%)	213 (49%)	432 (50%)
Male	215 (50%)	218 (51%)	433 (50%)
Other	0 (0%)	0 (0%)	0 (0%)
Child ethnicity^c^			
Asian^d^	17/434 (4%)	11/430 (3%)	28/864 (3%)
Black^e^	4/434 (<1%)	5/430 (1%)	9/864 (1%)
Mixed^f^	35/434 (8%)	34/430 (8%)	69/864 (8%)
White^g^	373/434 (86%)	378/430 (88%)	751/864 (87%)
Other^h^	4/434 (<1%)	0/430 (0%)	4/864 (<1%)
Prefer not to say	1/434 (<1%)	2/430 (<1%)	3/864 (<1%)
Child is fostered or adopted^c^			
Fostered	1/431 (<1%)	2/429 (<1%)	3/860 (<1%)
Adopted	3/431 (<1%)	8/429 (2%)	11/860 (1%)
Prefer not to say	2/431 (<1%)	2/429 (<1%)	4/860 (<1%)
Parent age, mean (*SD*) years^i^	36.7 (5.6)	37.1 (5.4)	36.9 (5.5)
Parent gender			
Female	409 (94%)	401 (93%)	810 (94%)
Male	25 (6%)	29 (7%)	54 (6%)
Other	0 (0%)	1 (<1%)	1 (<1%)
Parent relationship to child			
Mother	407 (94%)	396 (92%)	803 (93%)
Father	25 (6%)	29 (7%)	54 (6%)
Other	2 (<1%)	6 (1%)	8 (1%)
Parent ethnicity^c^			
Asian^d^	19/430 (4%)	14/428 (3%)	33/858 (4%)
Black^e^	6/430 (1%)	6/428 (1%)	12/858 (1%)
Mixed^f^	14/430 (3%)	11/428 (3%)	25/858 (3%)
White^g^	383/430 (89%)	392/428 (92%)	775/858 (90%)
Other^h^	6/430 (1%)	3/428 (1%)	9/858 (1%)
Prefer not to say	2/430 (<1%)	2/428 (<1%)	4/858 (<1%)
Parent is partnered	361 (83%)	353 (82%)	714 (83%)
Parent cohabits with partner^j^	346/361 (96%)	343/353 (97%)	689/714 (96%)
Number of children in household^c^			
None^k^	0/433 (0%)	1/429 (<1%)	1/862 (<1%)
One	93/433 (21%)	100/429 (23%)	193/862 (22%)
Two	245/433 (57%)	230/429 (54%)	475/862 (55%)
Three	72/433 (17%)	70/429 (16%)	142/862 (16%)
Four	15/433 (3%)	17/429 (4%)	32/862 (4%)
Five	4/433 (<1%)	9/429 (2%)	13/862 (2%)
Six	4/433 (<1%)	2/429 (<1%)	6/862 (<1%)
Type of housing^c^			
Privately rented	71/431 (16%)	81/427 (19%)	152/858 (18%)
Council or housing association rented	72/431 (17%)	62/427 (15%)	134/858 (16%)
Mortgage/owned	283/431 (66%)	277/427 (65%)	560/858 (65%)
Other	2/431 (<1%)	6/427 (1%)	8/858 (1%)
Prefer not to say	3/431 (1%)	1/427 (<1%)	4/858 (<1%)
Parent education			
School completion	22 (5%)	30 (7%)	52 (6%)
Further education	148 (34%)	145 (34%)	293 (34%)
Higher education	139 (32%)	134 (31%)	273 (32%)
Postgraduate education	113 (26%)	113 (26%)	226 (26%)
None of the above	5 (1%)	4 (1%)	9 (1%)
Prefer not to say	7 (2%)	5 (1%)	12 (1%)
Parent employment^c^			
Unemployed	35/432 (8%)	27/431 (6%)	62/863 (7%)
Part‐time	166/432 (38%)	173/431 (40%)	339/863 (39%)
Full time	145/432 (34%)	144/431 (33%)	289/863 (33%)
Student	12/432 (3%)	7/431 (2%)	19/863 (2%)
Retired	0/432 (0%)	1/431 (<1%)	1/863 (<1%)
Homemaker	54/432 (13%)	51/431 (12%)	105/863 (12%)
Other	16/432 (4%)	22/431 (5%)	38/863 (4%)
Prefer not to say	4/432 (1%)	6/431 (1%)	10/863 (1%)
Household annual income^c^			
<£16,000	62/419 (15%)	59/425 (14%)	121/844 (14%)
£16,001–£30,000	50/419 (12%)	56/425 (13%)	106/844 (13%)
£30,001–£40,000	38/419 (9%)	46/425 (11%)	84/844 (10%)
£40,001–£50,000	44/419 (11%)	44/425 (10%)	88/844 (10%)
£50,001–£60,000	47/419 (11%)	49/425 (12%)	96/844 (11%)
£60,001–£70,000	42/419 (10%)	29/425 (7%)	71/844 (8%)
£70,001–£80,000	29/419 (7%)	28/425 (7%)	57/844 (7%)
£80,001–£90,000	20/419 (5%)	29/425 (7%)	49/844 (6%)
£90,001–120,000	22/419 (5%)	26/425 (6%)	48/844 (6%)
>£120,000	21/419 (5%)	19/425 (4%)	40/844 (5%)
Prefer not to say	44/419 (11%)	40 /425 (9%)	84/844 (10%)
Index of Multiple Deprivation decile^l^			
1st	42 (10%)	42 (10%)	84 (10%)
2nd	40 (9%)	21 (5%)	61 (7%)
3rd	62 (14%)	47 (11%)	109 (13%)
4th	39 (9%)	29 (7%)	68 (8%)
5th	39 (9%)	71 (16%)	110 (13%)
6th	51 (12%)	43 (10%)	94 (11%)
7th	36 (8%)	49 (11%)	85 (10%)
8th	50 (12%)	31 (7%)	81 (9%)
9th	44 (10%)	60 (14%)	104 (12%)
10th	31 (7%)	38 (9%)	69 (8%)
Screening outcome			
One risk	247 (57%)	209 (48%)	456 (53%)
Screened positive for child anxiety only	117 (27%)	121 (28%)	238 (28%)
Screened positive for behavioural inhibition only	28 (6%)	11 (3%)	39 (5%)
Screened positive for parent anxiety only	102 (24%)	77 (18%)	179 (21%)
Two risks	149 (34%)	165 (38%)	314 (36%)
Screened positive for child anxiety symptoms + behavioural inhibition	53 (12%)	58 (13%)	111 (13%)
Screened positive for child anxiety symptoms + parent anxiety	89 (21%)	101 (23%)	190 (22%)
Screened positive for behavioural inhibition+parent anxiety	7 (2%)	6 (1%)	13 (2%)
Three risks	38 (9%)	57 (13%)	95 (11%)
Screened positive for child anxiety	297 (68%)	337 (78%)	634 (73%)
Screened positive for behavioural inhibition	126 (29%)	132 (31%)	258 (30%)
Screened positive for parent anxiety	236 (54%)	241 (56%)	477 (55%)
Baseline clinical measures			
PAS, mean (*SD*)	40.5 (17.2)	42.6 (16.4)	41.5 (16.8), ICC = 0.036
STSC Approach, mean (*SD*)	23.8 (7.6)	24.6 (7.8)	24.2 (7.7), ICC = 0.019
GAD‐7, mean (*SD*)	7.6 (5.0)	7.5 (5.1)	7.6 (5.0), ICC = 0.051
CALIS‐PV, mean (*SD*)	22.1 (13.2)	23.5 (13.5)	22.8 (13.4), ICC = 0.036
*SD*Q‐E, mean (*SD*)	8.1 (4.2)	8.7 (4.4)	8.4 (4.3), ICC = 0.011
POM, mean (*SD*)	35.3 (12.2)	35.8 (12.2)	35.6 (12.2), ICC = 0.048
PSCS Self‐efficacy, mean (*SD*)	27.9 (5.7)	27.6 (5.8)	27.7 (5.7), ICC = 0.024
RULES, mean (*SD*)	42.2 (14.6)	42.7 (13.5)	42.2 (14.1), ICC = 0.021
CQ‐P, mean (*SD*)	3.2 (1.4)	3.1 (1.4)	3.2 (1.4), ICC = 0
CAMP, mean (*SD*)	10.7 (5.5)	10.7 (5.6)	10.7 (5.6), ICC = 0.006

Data are *n* (%) or *n*/*N*(%), unless otherwise stated. Data were available for all randomised participants, unless otherwise stated. For all clinical measures, higher scores indicate worse functioning, with the exception of PSCS Self‐efficacy and CQ‐P where higher scores indicate better functioning. ICC, intra‐cluster (intra‐school) correlation coefficient; PAS, Pre‐school Anxiety Scale; STSC Approach, Approach subscale of the Short Temperament Scale for Children; GAD‐7, Generalised Anxiety Disorder Scale‐7; CALIS‐PV, Child Anxiety Life Interference Scale‐Preschool version; SDQ‐E, Strengths and Difficulties Questionnaire Externalising Scale; POM, Parent Overprotection Measure; PSCS Self‐efficacy, Self‐Efficacy Subscale of the Parenting Sense of Competence Scale; RULES, Responses to Uncertainty and Low Environmental Structure; CQ‐P, Coping Questionnaire; CAMP, Child Avoidance Measure.

^a^ Year group at screening.

^b^ Child age at randomisation.

^c^ Data were not available for all randomised participants.

^d^ Includes Asian or Asian British Indian, Pakistani, any other Asian background.

^e^ Includes Black or Black British African, Caribbean, any other Black background.

^f^ Includes White and Black Caribbean, White and Black African, White and Asian, any other mixed background.

^g^ Includes British, Irish, any other White background.

^h^ Includes Chinese.

^i^ Data were available for 381 in the Intervention arm and 385 in the Usual school practice arm.

^j^ Only includes those who are partnered.

^k^ 1 child in the Usual school practice arm lived with a second parent.

^l^ Based on family postcode: 1 = most deprived; 10 = least deprived.

Descriptive statistics for 6‐week outcomes are provided in Table S8 and group comparisons for 12‐week and 12‐month outcomes are provided in Tables 3 and 4.

**Table 3:** Main comparisons for 12 week secondary outcomes based on imputed data sets

	Intervention (*N* = 434)	Usual School Practice (*N* = 431)	Unadjusted			Adjusted			
	Mean (*SD*)	Mean (*SD*)	Mean difference	Standardised mean difference	Intra‐cluster correlation coefficient^a^	Mean difference (95% CI)	Standardised mean difference (95% CI)	*p* Value	Intra‐cluster correlation coefficient^a^
PAS	28.8 (17.5)	38.3 (17.1)	−8.8	−0.51	0.004	−7.3 (−9.1 to −5.5)	−0.43 (−0.53 to −0.32)	<.0001	0
STSC Approach	20.9 (7.4)	23.6 (7.7)	−2.5	−0.32	0.048	−1.9 (−2.5 to −1.2)	−0.24 (−0.33 to −0.16)	<.0001	0.014
GAD‐7	5.5 (4.8)	7.2 (5.6)	−1.6	−0.29	0.042	−1.8 (−2.3 to −1.2)	−0.31 (−0.42 to −0.21)	<.0001	0
CALIS‐PV	16.7 (13.0)	21.7 (15.2)	−4.8	−0.32	0.020	−3.8 (−5.3 to −2.4)	−0.25 (−0.35 to −0.16)	<.0001	0
SDQ‐E	6.9 (4.0)	8.1 (4.4)	−1.1	−0.25	0.004	−0.7 (−1.1 to −0.3)	−0.15 (−0.24 to −0.07)	.0006	0.013
POM	26.2 (12.5)	33.4 (12.3)	−6.2	−0.50	0.033	−5.8 (−7.0 to −4.5)	−0.47 (−0.57 to −0.37)	<.0001	0.008
PSCS Self‐efficacy	30.4 (5.6)	28.6 (5.9)	1.8	0.30	0.015	1.6 (1.0 to 2.3)	0.28 (0.17 to 0.39)	<.0001	0
RULES	36.9 (14.6)	41.1 (14.4)	−3.6	−0.25	0.029	−3.2 (−4.7 to −1.8)	−0.22 (−0.32 to −0.12)	<.0001	0
CQ‐P	4.5 (1.5)	3.7 (1.5)	0.7	0.46	0	0.7 (0.4 to 0.9)	0.44 (0.28 to 0.60)	<.0001	0
CAMP	9.0 (5.5)	10.6 (6.0)	−1.5	−0.25	0	−1.4 (−2.2 to −0.7)	−0.24 (−0.36 to −0.11)	.0003	0

For all outcomes, higher scores indicate worse functioning, with the exception of PSCS Self‐efficacy and CQ‐P where higher scores indicate better functioning. PAS, Pre‐school Anxiety Scale; STSC Approach, Approach subscale of the Short Temperament Scale for Children; GAD‐7, Generalised Anxiety Disorder Scale‐7; CALIS‐PV, Child Anxiety Life Interference Scale‐Preschool version; SDQ‐E, Strengths and Difficulties Questionnaire Externalising Scale; POM, Parent Overprotection Measure; PSCS Self‐efficacy, Self‐Efficacy Subscale of the Parenting Sense of Competence Scale; RULES, Responses to Uncertainty and Low Environmental Structure; CQ‐P, Coping Questionnaire; CAMP: Child Avoidance Measure.

^a^ Intra‐cluster (intra‐school) correlation coefficient estimates obtained from complete case analysis.

**Table 4:** Main comparisons for 12‐month outcomes based on imputed datasets

	Intervention (*N* = 434)	Usual School Practice (*N* = 431)	Unadjusted			Adjusted			
	*n*/*N* (%) or mean (*SD*)	*n*/*N* (%) or mean (*SD*)	Odds ratio or mean difference	Standardised mean difference	Intra‐cluster correlation coefficient^a^	Odd ratio or mean difference (95% CI)	Standardised mean difference (95% CI)	*p* Value	Intra‐cluster correlation coefficient^a^
Anxiety disorder diagnosis	21/310 (6.8%)	36/312 (11.5%)	0.71	..	0.034	0.67 (0.37 to 1.21)	..	.19	−0.009
PAS	26.4 (16.9)	33.3 (17.1)	−7.0	−0.41	0.013	−5.9 (−8.3 to −3.5)	−0.35 (−0.49 to −0.21)	<.0001	0
STSC Approach	20.1 (7.2)	21.6 (7.7)	−1.9	−0.25	0.031	−1.4 (−2.3 to −0.6)	−0.19 (−0.30 to −0.07)	.0012	0.004
GAD‐7	5.2 (4.4)	6.5 (5.1)	−1.3	−0.26	0.006	−1.4 (−2.0 to 0.8)	−0.28 (−0.40 to −0.15)	<.0001	0
CALIS‐PV	15.5 (13.8)	19.7 (15.7)	−4.7	−0.30	0.022	−4.1 (−6.0 to −2.1)	−0.26 (−0.38 to −0.14)	<.0001	0
SDQ‐E	6.6 (4.3)	8.1 (4.9)	−1.5	−0.30	0	−1.0 (−1.6 to −0.5)	−0.21 (−0.32 to −0.10)	.0002	0.002
POM	24.9 (11.5)	30.7 (11.8)	−5.3	−0.45	0.019	−4.8 (−6.3 to −3.4)	−0.41 (−0.53 to −0.29)	<.0001	0
PSCS Self‐efficacy	30.4 (5.4)	29.0 (5.9)	1.4	0.24	0	1.3 (0.5 to 2.1)	0.22 (0.09 to 0.36)	.0011	0.007
RULES	36.1 (15.2)	39.9 (15.4)	−4.6	−0.29	0	−4.4 (−6.3 to −2.5)	−0.28 (−0.41 to −0.16)	<.0001	0
CQ‐P	4.9 (1.6)	4.2 (1.6)	0.7	0.42	0.017	0.7 (0.4 to 0.9)	0.40 (0.23 to 0.57)	<.0001	0.020
CAMP	8.0 (5.8)	9.9 (6.0)	−1.8	−0.29	0.017	−1.9 (−2.8 to −0.9)	−0.31 (−0.47 to −0.15)	.0002	0.029

For all outcomes, higher scores indicate worse functioning, with the exception of PSCS Self‐efficacy and CQ‐P, where higher scores indicate better functioning. PAS, Pre‐school Anxiety Scale; STSC Approach, Approach subscale of the Short Temperament Scale for Children; GAD‐7, Generalised Anxiety Disorder Scale‐7; CALIS‐PV, Child Anxiety Life Interference Scale‐Preschool version; SDQ‐E, Strengths and Difficulties Questionnaire Externalising Scale; POM, Parent Overprotection Measure; PSCS Self‐efficacy, Self‐Efficacy Subscale of the Parenting Sense of Competence Scale; RULES, Responses to Uncertainty and Low Environmental Structure; CQ‐P, Coping Questionnaire; CAMP, Child Avoidance Measure. ".." Not Applicable.

^a^ Intra‐cluster (intra‐school) correlation coefficient estimates obtained from complete case analysis.

The number of children with an anxiety disorder diagnosis at 12‐month follow‐up was lower than expected across both arms: 21 out of 310 (6.8%) assessed children in the intervention arm compared to 36 out of 312 (11.5%) in the usual school arm. In the primary analysis using imputed data sets, there was no statistically significant difference between groups in the presence of an anxiety disorder diagnosis (adjusted odds ratio 0.67 [0.37 to 1.21], *p* = .19) (Table 4). For the complete case analysis, the confidence intervals were narrower and the difference between arms was statistically significant (adjusted odds ratio 0.57 [0.34 to 0.96], *p* = .034) (Table 5). Findings from the secondary CACE analysis for 12‐month anxiety disorder status were similar to the intention‐to‐treat analysis (adjusted odds ratio 0.54 [0.24 to 1.27] based on imputed datasets and 0.53 [0.26 to 0.84] for complete cases). Sensitivity analyses only including those who completed the diagnostic assessment within the 12‐week data collection window also followed the same pattern (adjusted odds ratio 0.65 [0.36 to 1.17], *p* = .15 based on imputed datasets; 0.54 [0.32 to 0.90], *p* = .018 for complete cases).

**Table 5:** Effects on anxiety disorder diagnosis at 12 months for primary and secondary analyses

	Analysis based on imputed datasets			Complete case analysis		
	Unadjusted	Adjusted		Unadjusted	Adjusted	
	Odds ratio	Odds ratio (95% CI)	*p* Value	Odds ratio	Odds ratio (95% CI)	*p* Value
Intention‐to‐treat analysis	0.71	0.67 (0.37 to 1.21)	.19	0.61	0.57 (0.34 to 0.96)	.034
Sensitivity analysis: CACE analysis	0.59	0.54 (0.24 to 1.27)	.16	0.57	0.53 (0.26 to 0.84)^a^	
Sensitivity analysis: data provided within data collection window	0.70	0.65 (0.36 to 1.17)	.15	0.59	0.54 (0.32 to 0.90)	.018

CACE: complier average causal effect.

^a^ *p* Value cannot be calculated for CACE analysis when using complete case data.

In contrast, there were statistically significant differences between groups in favour of the intervention arm in the main analysis using imputed datasets across all 10 secondary clinical outcomes at both 12 weeks and 12 months (Tables 3 and 4). The absolute values for the standardised mean differences for secondary clinical outcomes ranged from 0.15 to 0.47 at 12 weeks and 0.19 to 0.41 at 12 months. Complete case analysis of 12‐week and 12‐month secondary outcomes showed the same pattern as primary analysis of imputed data for these outcomes (Tables S9 and S10).

Summary statistics and exemplar quotes from the acceptability questionnaire responses are provided in Tables S11–S18. Participants across both arms were positive about taking part. More than 90% of intervention arm respondents (321 out of 340 [94%] to 328 out of 338 [97%] at 12 weeks, and 273 out of 299 [91%] to 283 out of 299 [95%] at 12 months) agreed or completely agreed with statements describing positive experiences of the website and support calls.

No serious adverse events were recorded. One adverse event related to trial procedures was recorded in each arm (details provided in Table S19). On acceptability questionnaires, five participants (4 in the usual school practice arm and 1 in the intervention arm) reported some expected levels of distress or discomfort completing trial questionnaires, and four intervention arm participants reported experiencing some mild expected level of distress or discomfort related to participating in the intervention.

## Discussion

This trial compared parent‐led CBT delivered via online and telephone support alongside usual school provision with usual school provision only for young children identified as at risk for anxiety disorders through a universal offer of screening in schools. We found that while the difference in the frequency of diagnosable anxiety disorders at 12 months was not statistically significant, there were statistically significant differences favouring the intervention on all other assessed clinical outcomes. The intervention reduced child anxiety symptoms, known risks for future anxiety disorders, anxiety‐related inference, externalising symptoms, and changed parent and child behaviours targeted by cognitive behavioural interventions. Parent feedback highlighted positive experiences of the intervention and no serious adverse events or adverse events directly related to the intervention were reported. These findings demonstrate the potential for brief CBT interventions delivered remotely for parents of at‐risk young children identified through screening in schools to provide an efficient way to alter the trajectory of impairing anxiety.

Diagnosable anxiety disorders at 12‐month follow‐up were notably lower across both arms than we expected (6.8% and 11.5% in the intervention and usual school practice arm respectively, compared to 50% that was assumed for the control arm). We used a novel approach to recruitment that incorporated a universal offer of screening for multiple risks in young children and evaluated providing support in the context of any one of these risks. It is plausible that this approach contributed to the lower rate of anxiety disorders at 12 months than reported in trials that selected young children solely on the basis of behavioural inhibition. Behavioural inhibition is associated with a threefold increased risk in future anxiety disorders (Sandstrom et al., 2020) so it is notable that in our sample this was the least frequent risk overall (30% screened positive for behavioural inhibition, compared to 73% with elevated anxiety symptoms and 55% with elevated parent anxiety) and the least frequent single risk (5% screened positive for behavioural inhibition only, compared to 28% for elevated anxiety only and 21% for parent anxiety only). Where young children have previously been identified through screening for inhibition in preschools and randomly allocated to parent‐led CBT delivered in groups or usual care, 44% and 50%, respectively, had diagnosable anxiety disorders at 12 months (a difference that was not statistically significant) (Bayer et al., 2018), and where inhibited children recruited through wider advertisements were randomised to a self‐guided online parent‐led CBT programme or a wait‐list, 40% and 54%, respectively, had diagnosable anxiety disorders at 6 months (a difference that was statistically significant) (Morgan et al., 2017). We judged a 15 percentage point reduction in diagnosable anxiety disorders to be meaningful where the expected prevalence rate for a sample recruited through screening in schools/preschools is 50% but our findings do raise questions about what should be considered a meaningful reduction in diagnosable anxiety disorders over 12 months for a wider population where fewer children may develop disorder‐level difficulties during this period. To detect the same relative reduction in diagnosable anxiety disorders as we originally planned but for a wider population with a much lower expected prevalence (e.g. 10% to 7% rather than 50% to 35%), we would have needed a much larger sample size. The observed prevalence rate does also raise questions about whether adjustments to the selection criteria should be considered in order to prioritise intervention for a narrower population at greater risk of disorder‐level difficulties over a 12‐month period. It will be beneficial for future research to consider the pros and cons of alternative recruitment approaches (e.g. screening for behavioural inhibition only, screening for multiple risks with adjusted cut‐points) and track longer‐term intervention outcomes for children with different combinations and severity of risks at screening.

The positive intervention effects across all secondary clinical outcomes point to potential longer‐term benefits of providing parent‐led CBT via online and telephone support for young children identified as being at risk for anxiety disorders on the basis of any one of the three assessed risks. The reduction in all three risk factors as well as parent overprotective behaviour, which is predicted to play a key role in the development and maintenance of anxiety disorders (Rapee, Creswell, Kendall, Pine, & Waters, 2023), is promising for protection against future anxiety disorders. Notably, interventions that specifically target inhibited children have not previously been found to reduce parent anxiety or parent overprotection (Bayer et al., 2018; Morgan et al., 2017), so these may be particular strengths of this intervention. Completion rates for the intervention (76% completed the core intervention content, 73% completed the full content) also illustrate a potential advantage over previously evaluated delivery formats (34% attended most in‐person group sessions (Bayer et al., 2018); 25% completed all online content for a predominantly self‐guided online intervention (Morgan et al., 2017)). Parent feedback illustrates that the regular but modest amount of therapist contact (mean of 2.5 hr per family) was perceived as important for maintaining engagement.

The inclusion of a diverse range of schools with different characteristics is a strength of this study and resulted in a sample with a range of sociodemographic characteristics. The participation rate in screening was lower than originally expected (15% compared to 50%), although the fact that 50% of those screened were eligible indicates some degree of self‐selection, with those more likely to be eligible also more likely to participate. There was, however, marked variation in participation in screening and trial enrolment across schools. Fewer children were screened and enrolled in schools with above‐average levels of deprivation (a mean of 18 screened and 7 enrolled) than below average (a mean of 30 screened and 11 enrolled). Characteristics of eligible families who did and did not enrol showed that families who lived in rented housing, parents with lower levels of education, unemployed parents, families from minoritised ethnic backgrounds and single parents were over‐represented in those who did not enrol. It will be important that future research and implementation efforts prioritise working collaboratively with schools and families to determine how best to address barriers to participation for each of these groups and in schools with higher levels of deprivation.

The study had several limitations. Diagnostic outcomes were assessed and assigned by individuals who were blind to trial arm, but it was not possible for participants or study team members who recruited participants and collected other study data to be blind to trial arm. Secondary clinical outcomes were assessed using parent‐report questionnaires which means there is a risk of bias from parents. Relying on a single reporter for child mental health assessments is also not optimal, although it is typical for children under 8 years, where there is a lack of validated child self‐report measures. Retention to 12‐month follow‐up was slightly below our original target of 80%, with only 72% completing the diagnostic assessment and 78% at least one 12‐month outcome. These retention rates were similar across trial arms, but differences in demographic and clinical characteristics between those with and without diagnostic outcome data introduce a source of bias that needs to be considered. In the complete case analysis, there was a statistically significant difference in the presence of anxiety disorders at 12 months favouring the intervention arm, but we consider this result to be less robust than our primary analysis in which missing data were imputed. In contrast, for secondary questionnaire outcomes we found consistent results across analyses using imputed data and complete cases, which means we can be confident in the robustness of these results. Finally, as a pragmatic trial, there was no restriction on the mental health support provided in schools or accessed by families during the trial period. Encouragingly, support provided by schools did not differ by trial arm, but the perceived helpfulness of taking part in the trial for their family among parents in the usual school practice arm was notable, which suggests there could be some benefit to completing trial measures and contact with the study team that would not be present in real world ‘usual practice’.

Although we did not find evidence of an intervention effect on diagnostic outcomes at 12 months, this study provides robust evidence that CBT delivered via online and telephone support for parents of young children identified as at risk for anxiety disorders on the basis of elevated anxiety symptoms, inhibition and/or parent anxiety brings benefits for children and families across a broad range of other outcomes. Delivery of this efficient and accessible intervention for young children identified through screening in schools has the potential to provide long‐term protection against future anxiety disorders at scale. Efforts need to now focus on establishing optimal screening criteria, investigating longer‐term outcomes and determining how best to successfully implement this approach through schools in a way that maximises accessibility for all families who are likely to benefit.

## Ethical considerations

Ethical approval was obtained from the University of Oxford Medical Sciences Interdivisional Research Ethics Committee (Reference: R62531; Approval date: 6 Jan 2021). Parents/carers provided written informed consent to participate in screening and the trial.

## Trial registration

The study was prospectively registered on 14th January 2021 on ISRCTN (82398107) https://www.isrctn.com/ISRCTN82398107.

**Key points**

**What's known?**

- Screening in schools to identify young children with one or more known risks for anxiety disorders and offering them accessible CBT could maximise reach for effective early intervention and prevention.

**What's new?**

- To our knowledge, this is the first trial to evaluate providing parent‐led CBT via online and telephone support for young children identified as having at least one risk for future anxiety disorders through screening in schools.
- The frequency of anxiety disorders at 12 months was relatively low and the intervention was not associated with a significant reduction compared to usual school provision, but it did reduce anxiety symptoms and related interference and alter additional risks and intervention targets.

**What's relevant?**

- Findings indicate that implementing this approach has the potential to improve a broad range of outcomes for young children and reduce known risks for future anxiety disorders.

## Supporting information


**Appendix S1.** Measure of social, emotional and mental health and wellbeing provision in schools.
**Appendix S2.** Summary of criteria used to assess school social, emotional, mental health and wellbeing provision.
**Appendix S3.** Minor adaptations to content of OSI for the MYCATS Trial.
**Appendix S4.** Secondary clinical outcomes.
**Table S1.** Number (%) of children screened and enrolled on the trial according to school‐level deprivation.
**Table S2.** Summary of characteristics for children eligible for the trial according to enrolment status.
**Table S3.** Completion of OSI online modules and support calls in the Intervention arm (*N* = 434).
**Table S4.** Therapist and supervisor time (minutes) spent on intervention and supervision for OSI users.
**Table S5.** Provision of follow‐up data by trial arm status.
**Table S6.** Summary of demographic characteristics by trial arm status and provision primary outcome data.
**Table S7.** Summary (mean (*SD*)) of outcomes at baseline by trial arm status and provision of primary outcome data.
**Table S8.** Summary of outcomes at 6‐weeks.
**Table S9.** Analysis of secondary outcomes at 12‐weeks based on complete case analysis.
**Table S10.** Analysis of outcomes at 12‐months based on complete case analysis.
**Table S11.** Summary of responses to closed questions on the baseline acceptability questionnaire.
**Table S12.** Summary of participant feedback provided in free text responses on baseline acceptability questionnaire.
**Table S13.** Summary of responses to closed questions on the 12‐week acceptability questionnaire.
**Table S14.** Summary participant feedback provided in free text responses on 12‐week acceptability questionnaire (intervention arm, *N* = 342).
**Table S15.** Summary participant feedback provided in free text responses on 12‐week acceptability questionnaire (Usual school practice arm, *N* = 355).
**Table S16.** Summary of responses to closed questions on the 12‐month acceptability questionnaire.
**Table S17.** Summary participant feedback provided in free text responses on 12‐month acceptability questionnaire (intervention arm, *N* = 301).
**Table S18.** Summary participant feedback provided in free text responses on 12‐month acceptability questionnaire (Usual school practice arm, *N* = 308).
**Table S19.** Reported Adverse Events.

## Data Availability

Deidentified individual participant data, a data dictionary and the analysis code will be made available on an open access data repository accompanied by the study protocol and the statistical analysis plan as soon as possible after publication; for more information, contact the corresponding author.

## References

[jcpp70119-bib-0001] Bayer, J.K. , Beatson, R. , Bretherton, L. , Hiscock, H. , Wake, M. , Gilbertson, T. , … & Rapee, R.M. (2018). Translational delivery of Cool Little Kids to prevent child internalising problems: Randomised controlled trial. Australian and New Zealand Journal of Psychiatry, 52, 181–191. 28831814 10.1177/0004867417726582

[jcpp70119-bib-0002] Bayer, J.K. , Prendergast, L.A. , Brown, A. , Harris, L. , Bretherton, L. , Hiscock, H. , … & Rapee, R.M. (2021). Cool Little Kids translational trial to prevent internalising: Two‐year outcomes and prediction of parent engagement. Child and Adolescent Mental Health, 26, 211–219. 33247555 10.1111/camh.12420

[jcpp70119-bib-0003] Caldwell, D.M. , Davies, S.R. , Hetrick, S.E. , Palmer, J.C. , Caro, P. , López‐López, J.A. , … & Welton, N.J. (2019). School‐based interventions to prevent anxiety and depression in children and young people: A systematic review and network meta‐analysis. The Lancet Psychiatry, 6, 1011–1020. 31734106 10.1016/S2215-0366(19)30403-1 PMC7029281

[jcpp70119-bib-0004] Children's Commissioner . (2024). Children's mental health services 2022–23. Available from: https://assets.childrenscommissioner.gov.uk/wpuploads/2024/03/Childrens‐mental‐health‐services‐22‐23_CCo‐final‐report.pdf

[jcpp70119-bib-0005] Chronis‐Tuscano, A. , Novick, D.R. , Danko, C.M. , Smith, K.A. , Wagner, N.J. , Wang, C.H. , … & Rubin, K.H. (2022). Early intervention for inhibited young children: A randomized controlled trial comparing the Turtle Program and Cool Little Kids. Journal of Child Psychology and Psychiatry, 63, 273–281. 34184792 10.1111/jcpp.13475 PMC11270476

[jcpp70119-bib-0006] Crane, M.E. , & Kendall, P.C. (2020). Psychometric evaluation of the child and parent versions of the coping questionnaire. Child Psychiatry & Human Development, 51, 709–720. 32157488 10.1007/s10578-020-00975-w PMC7483227

[jcpp70119-bib-0007] Creswell, C. , Nauta, M.H. , Hudson, J.L. , March, S. , Reardon, T. , Arendt, K. , … & Kendall, P.C. (2021). Research review: Recommendations for reporting on treatment trials for child and adolescent anxiety disorders–an international consensus statement. Journal of Child Psychology and Psychiatry, 62, 255–269. 32683742 10.1111/jcpp.13283

[jcpp70119-bib-0008] Creswell, C. , Taylor, L. , Giles, S. , Howitt, S. , Radley, L. , Whitaker, E. , … & Yu, L.‐M. (2024). Digitally augmented, parent‐led CBT versus treatment as usual for child anxiety problems in child mental health services in England and Northern Ireland: A pragmatic, non‐inferiority, clinical effectiveness and cost‐effectiveness randomised controlled trial. The Lancet Psychiatry, 11, 193–209. 38335987 10.1016/S2215-0366(23)00429-7

[jcpp70119-bib-0009] Creswell, C. , Violato, M. , Fairbanks, H. , White, E. , Parkinson, M. , Abitabile, G. , … & Cooper, P.J. (2017). Clinical outcomes and cost‐effectiveness of brief guided parent‐delivered cognitive behavioural therapy and solution‐focused brief therapy for treatment of childhood anxiety disorders: A randomised controlled trial. The Lancet Psychiatry, 4, 529–539. 28527657 10.1016/S2215-0366(17)30149-9 PMC5483485

[jcpp70119-bib-0010] Fusar‐Poli, P. , Correll, C.U. , Arango, C. , Berk, M. , Patel, V. , & Ioannidis, J.P.A. (2021). Preventive psychiatry: A blueprint for improving the mental health of young people. World Psychiatry, 20, 200–221. 34002494 10.1002/wps.20869 PMC8129854

[jcpp70119-bib-0011] Gilbertson, T.J. , Morgan, A.J. , Rapee, R.M. , Lyneham, H.J. , & Bayer, J.K. (2017). Psychometric properties of the child anxiety life interference scale – Preschool version. Journal of Anxiety Disorders, 52, 62–71. 29053989 10.1016/j.janxdis.2017.10.002

[jcpp70119-bib-0012] Ginsburg, G.S. , Drake, K.L. , Tein, J.Y. , Teetsel, R. , & Riddle, M.A. (2015). Preventing onset of anxiety disorders in offspring of anxious parents: A randomized controlled trial of a family‐based intervention. American Journal of Psychiatry, 172, 1207–1214. 26404420 10.1176/appi.ajp.2015.14091178 PMC6013063

[jcpp70119-bib-0013] Goodman, A. , & Goodman, R. (2009). Strengths and difficulties questionnaire as a dimensional measure of child mental health. Journal of the American Academy of Child and Adolescent Psychiatry, 48, 400–403. 19242383 10.1097/CHI.0b013e3181985068

[jcpp70119-bib-0014] Grund, S. , Ludtke, O. , & Robitzsch, A. (2016). Multiple imputation of multilevel missing data: An introduction to the R package pan. Sage Open, 6, 4.

[jcpp70119-bib-0015] Harris, P.A. , Taylor, R. , Minor, B.L. , Elliott, V. , Fernandez, M. , O'Neal, L. , … & Duda, S.N. (2019). The REDCap consortium: Building an international community of software platform partners. Journal of Biomedical Informatics, 95, 103208. 31078660 10.1016/j.jbi.2019.103208 PMC7254481

[jcpp70119-bib-0016] Hill, C. , Reardon, T. , Taylor, L. , & Creswell, C. (2022). Online Support and Intervention for Child Anxiety (OSI): Development and usability testing. JMIR Formative Research, 6, e29846. 35416781 10.2196/29846 PMC9047721

[jcpp70119-bib-0017] Howes Vallis, E. , Zwicker, A. , Uher, R. , & Pavlova, B. (2020). Cognitive‐behavioural interventions for prevention and treatment of anxiety in young children: A systematic review and meta‐analysis. Clinical Psychology Review, 81, 101904. 32891925 10.1016/j.cpr.2020.101904

[jcpp70119-bib-0018] James, A.C. , Reardon, T. , Soler, A. , James, G. , & Creswell, C. (2020). Cognitive behavioural therapy for anxiety disorders in children and adolescents. Cochrane Database of Systematic Reviews, 11, CD013162. 33196111 10.1002/14651858.CD013162.pub2 PMC8092480

[jcpp70119-bib-0019] Johnston, C. , & Mash, E.J. (1989). A measure of parenting satisfaction and efficacy. Journal of Clinical Child Psychology, 18, 167–175.

[jcpp70119-bib-0020] Jones, B.G. , Reardon, T. , Creswell, C. , Dodd, H.F. , Hill, C. , Jasper, B. , … & Ukoumunne, O.C. (2022). Minimising Young Children's Anxiety through Schools (MY‐CATS): Statistical analysis plan for a cluster randomised controlled trial to evaluate the effectiveness and cost‐effectiveness of an online parent‐led intervention compared with usual school practice for young children identified as at risk for anxiety disorders. Trials, 23, 1054. 36575433 10.1186/s13063-022-06899-1 PMC9795669

[jcpp70119-bib-0021] Kennedy, S.J. , Rapee, R.M. , & Edwards, S.L. (2009). A selective intervention program for inhibited preschool‐aged children of parents with an anxiety disorder: Effects on current anxiety disorders and temperament. Journal of the American Academy of Child & Adolescent Psychiatry, 48, 602–609. 19454916 10.1097/CHI.0b013e31819f6fa9

[jcpp70119-bib-0022] Kreuze, L.J. , Pijnenborg, G.H.M. , De Jonge, Y.B. , & Nauta, M.H. (2018). Cognitive‐behavior therapy for children and adolescents with anxiety disorders: A meta‐analysis of secondary outcomes. Journal of Anxiety Disorders, 60, 43–57. 30447493 10.1016/j.janxdis.2018.10.005

[jcpp70119-bib-0023] Kroenke, K. , Spitzer, R.L. , Williams, J.B. , Monahan, P.O. , & Löwe, B. (2007). Anxiety disorders in primary care: Prevalence, impairment, comorbidity, and detection. Annals of Internal Medicine, 146, 317–325. 17339617 10.7326/0003-4819-146-5-200703060-00004

[jcpp70119-bib-0024] Lawrence, P.J. , Harvey, K. , Williams, C. , & Creswell, C. (2022). Barriers and facilitators to targeted anxiety prevention programmes in families at risk: A qualitative interview study. European Child & Adolescent Psychiatry, 31, 565–575. 33346882 10.1007/s00787-020-01703-4 PMC9034995

[jcpp70119-bib-0025] Lawrence, P.J. , Murayama, K. , & Creswell, C. (2019). Systematic review and meta‐analysis: Anxiety and depressive disorders in offspring of parents with anxiety disorders. Journal of the American Academy of Child and Adolescent Psychiatry, 58, 46–60. 30577938 10.1016/j.jaac.2018.07.898

[jcpp70119-bib-0026] Lyneham, H.J. , & Rapee, R.M. (2005). Agreement between telephone and in‐person delivery of a structured interview for anxiety disorders in children. Journal of the American Academy of Child & Adolescent Psychiatry, 44, 274–282. 15725972 10.1097/00004583-200503000-00012

[jcpp70119-bib-0027] Mifsud, C. , & Rapee, R.M. (2005). Early intervention for childhood anxiety in a school setting: Outcomes for an economically disadvantaged population. Journal of the American Academy of Child and Adolescent Psychiatry, 44, 996–1004. 16175104 10.1097/01.chi.0000173294.13441.87

[jcpp70119-bib-0028] Morgan, A.J. , Rapee, R.M. , Salim, A. , Goharpey, N. , Tamir, E. , McLellan, L.F. , & Bayer, J.K. (2017). Internet‐delivered parenting program for prevention and early intervention of anxiety problems in young children: Randomized controlled trial. Journal of the American Academy of Child and Adolescent Psychiatry, 56, 417–425. 28433091 10.1016/j.jaac.2017.02.010

[jcpp70119-bib-0029] Office for National Statistics . (2023). Employee earnings in the UK: 2023. Available from: https://www.ons.gov.uk/employmentandlabourmarket/peopleinwork/earningsandworkinghours/bulletins/annualsurveyofhoursandearnings/2023

[jcpp70119-bib-0030] Parker, K. , Nunns, M. , Xiao, Z. , Ford, T. , Stallard, P. , Kuyken, W. , … & Ukoumunne, O.C. (2025). Patterns of intra‐cluster correlation coefficients in school‐based cluster randomised controlled trials of interventions for improving social‐emotional functioning outcomes in pupils: A secondary data analysis of five UK‐based studies. BMC Medical Research Methodology, 25, 120. 40319234 10.1186/s12874-025-02574-6 PMC12048950

[jcpp70119-bib-0031] Plummer, F. , Manea, L. , Trepel, D. , & McMillan, D. (2016). Screening for anxiety disorders with the GAD‐7 and GAD‐2: A systematic review and diagnostic metaanalysis. General Hospital Psychiatry, 39, 24–31. 26719105 10.1016/j.genhosppsych.2015.11.005

[jcpp70119-bib-0032] Pollard, J. , Reardon, T. , Williams, C. , Creswell, C. , Ford, T. , Gray, A. , … & Violato, M. (2023). The multifaceted consequences and economic costs of child anxiety problems: A systematic review and meta‐analysis. JCPP Advances, 3, e12149. 37720587 10.1002/jcv2.12149 PMC10501703

[jcpp70119-bib-0033] R Core Team . (2020). R: A language and environment for statistical computing. Vienna, Austria: R Foundation for Statistical Computing.

[jcpp70119-bib-0034] Rapee, R.M. , Abbott, M.J. , & Lyneham, H.J. (2006). Bibliotherapy for children with anxiety disorders using written materials for parents: A randomized controlled trial. Journal of Consulting and Clinical Psychology, 74, 436–444. 16822101 10.1037/0022-006X.74.3.436

[jcpp70119-bib-0035] Rapee, R.M. , Creswell, C. , Kendall, P.C. , Pine, D.S. , & Waters, A.M. (2023). Anxiety disorders in children and adolescents: A summary and overview of the literature. Behaviour Research and Therapy, 168, 104376. 37499294 10.1016/j.brat.2023.104376

[jcpp70119-bib-0036] Rapee, R.M. , Edwards, S.L. , Mabood, S. , & Freeman, J.Y.A. (2024). Psychometric properties of a self‐report measure of overprotective parenting: The parental overprotection measure (POM). Child Psychiatry & Human Development. Advance online publication. 10.1007/s10578-024-01801-3 39704970

[jcpp70119-bib-0037] Rapee, R.M. , Kennedy, S. , Ingram, M. , Edwards, S. , & Sweeney, L. (2005). Prevention and early intervention of anxiety disorders in inhibited preschool children. Journal of Consulting and Clinical Psychology, 73, 488. 15982146 10.1037/0022-006X.73.3.488

[jcpp70119-bib-0038] Reardon, T. , Dodd, H. , Hill, C. , Jasper, B. , Lawrence, P.J. , Morgan, F. , … & Creswell, C. (2022). Minimising young children's anxiety through schools (MY‐CATS): Protocol for a cluster randomised controlled trial to evaluate the effectiveness and cost‐effectiveness of an online parent‐led intervention compared with usual school practice for young children identified as at risk for anxiety disorders. Trials, 23, 149. 35168635 10.1186/s13063-022-06010-8 PMC8848959

[jcpp70119-bib-0039] Reardon, T. , Harvey, K. , & Creswell, C. (2020). Seeking and accessing professional support for child anxiety in a community sample. European Child & Adolescent Psychiatry, 29, 649–664. 31410579 10.1007/s00787-019-01388-4 PMC7250799

[jcpp70119-bib-0040] Reardon, T. , Ukoumunne, O.C. , Ball, S. , Brown, P. , Ford, T. , Gray, A. , & Creswell, C. (2025). Development of a brief assessment tool to identify children with probable anxiety disorders. JCPP Advances, 5, e12265. 40519956 10.1002/jcv2.12265 PMC12159304

[jcpp70119-bib-0041] Sanchez, A.L. , Cornacchio, D. , Chou, T. , Leyfer, O. , Coxe, S. , Pincus, D. , & Comer, J.S. (2017). Development of a scale to evaluate young children's responses to uncertainty and low environmental structure. Journal of Anxiety Disorders, 45, 17–23. 27907833 10.1016/j.janxdis.2016.11.006

[jcpp70119-bib-0042] Sandstrom, A. , Uher, R. , & Pavlova, B. (2020). Prospective association between childhood behavioral inhibition and anxiety: A meta‐analysis. Research on Child and Adolescent Psychopathology, 48, 57–66. 10.1007/s10802-019-00588-5 31642030

[jcpp70119-bib-0043] Sanson, A. , Pedlow, R. , Cann, W. , Prior, M. , & Oberklaid, F. (1996). Shyness ratings: Stability and correlates in early childhood. International Journal of Behavioral Development, 19, 705–724.

[jcpp70119-bib-0044] Silverman, W.K. , & Albano, A.M. (1996). The Anxiety Disorders Interview Schedule for DSM‐IV – Child and Parent Versions. San Antonio, TX: Psychological Corporation.

[jcpp70119-bib-0045] Solmi, M. , Radua, J. , Olivola, M. , Croce, E. , Soardo, L. , Salazar de Pablo, G. , … & Fusar‐Poli, P. (2022). Age at onset of mental disorders worldwide: Large‐scale meta‐analysis of 192 epidemiological studies. Molecular Psychiatry, 27, 281–295. 34079068 10.1038/s41380-021-01161-7 PMC8960395

[jcpp70119-bib-0046] Spence, S.H. , Rapee, R. , McDonald, C. , & Ingram, M. (2001). The structure of anxiety symptoms among preschoolers. Behaviour Research and Therapy, 39, 1293–1316. 11686265 10.1016/s0005-7967(00)00098-x

[jcpp70119-bib-0047] Spitzer, R.L. , Kroenke, K. , Williams, J.B.W. , & Löwe, B. (2006). A brief measure for assessing generalized anxiety disorder: The GAD‐7. Archives of Internal Medicine, 16, 1092–1097. 10.1001/archinte.166.10.1092 16717171

[jcpp70119-bib-0048] Stallard, P. , Skryabina, E. , Taylor, G. , Phillips, R. , Daniels, H. , Anderson, R. , & Simpson, N. (2014). Classroom‐based cognitive behaviour therapy (FRIENDS): A cluster randomised controlled trial to Prevent Anxiety in Children through Education in Schools (PACES). The Lancet Psychiatry, 1, 185–192. 26360730 10.1016/S2215-0366(14)70244-5

[jcpp70119-bib-0049] StatCorp . (2022). Stata statistical software: Release 18. College Station, TX: StatCorp.

[jcpp70119-bib-0050] Steinsbekk, S. , Ranum, B. , & Wichstrøm, L. (2022). Prevalence and course of anxiety disorders and symptoms from preschool to adolescence: A 6‐wave community study. Journal of Child Psychology and Psychiatry, 63, 527–534. 34318492 10.1111/jcpp.13487

[jcpp70119-bib-0051] UK Government . (2019). Schools, pupils and their characteristics: January 2019. Available from: https://www.gov.uk/government/statistics/schools‐pupils‐and‐their‐characteristics‐january‐2019

[jcpp70119-bib-0052] UK Government . (2022). Population of England and Wales. https://www.ethnicity‐facts‐figures.service.gov.uk/uk‐population‐by‐ethnicity/national‐and‐regional‐populations/population‐of‐england‐and‐wales/latest/

[jcpp70119-bib-0053] Whiteside, S.P.H. , Gryczkowski, M. , Ale, C.M. , Brown‐Jacobsen, A.M. , & McCarthy, D.M. (2013). Development of child‐ and parent‐report measures of behavioral avoidance related to childhood anxiety disorders. Behavior Therapy, 44, 325–337. 23611081 10.1016/j.beth.2013.02.006

