# Supplementary material for: Parent‐led CBT delivered via online and telephone support alongside usual school practice versus usual school practice only for young children identified as at risk for anxiety disorders through screening in schools: a cluster randomised controlled trial
Source: J Child Psychol Psychiatry. 2026 Feb 19;67(8):1280–96. doi: 10.1111/jcpp.70119 (PMC13341351; doi:10.1111/jcpp.70119)
Supplement: Supplementary file 1 — Appendix S1. Measure of social, emotional and mental health and wellbeing provision in schools. Appendix S2. Summary of criteria used to assess school social, emotional, mental health and wellbeing provision. Appendix S3. Minor adaptations to content of OSI for the MYCATS Trial. Appendix S4. Secondary clinical outcomes. Table S1. Number (%) of children screened and enrolled on the trial according to school‐level deprivation. Table S2. Summary of characteristics for children eligible for the trial according to enrolment status. Table S3. Completion of OSI online modules and support calls in the Intervention arm (N = 434). Table S4. Therapist and supervisor time (minutes) spent on intervention and supervision for OSI users. Table S5. Provision of follow‐up data by trial arm status. Table S6. Summary of demographic characteristics by trial arm status and provision primary outcome data. Table S7. Summary (mean (SD)) of outcomes at baseline by trial arm status and provision of primary outcome data. Table S8. Summary of outcomes at 6‐weeks. Table S9. Analysis of secondary outcomes at 12‐weeks based on complete case analysis. Table S10. Analysis of outcomes at 12‐months based on complete case analysis. Table S11. Summary of responses to closed questions on the baseline acceptability questionnaire. Table S12. Summary of participant feedback provided in free text responses on baseline acceptability questionnaire. Table S13. Summary of responses to closed questions on the 12‐week acceptability questionnaire. Table S14. Summary participant feedback provided in free text responses on 12‐week acceptability questionnaire (intervention arm, N = 342). Table S15. Summary participant feedback provided in free text responses on 12‐week acceptability questionnaire (Usual school practice arm, N = 355). Table S16. Summary of responses to closed questions on the 12‐month acceptability questionnaire. Table S17. Summary participant feedback provided in free text responses on 12‐month accept [file JCPP-67-1280-s001.docx]

**Parent-led CBT delivered via online and telephone support alongside usual school practice versus usual school practice only for young children identified as at risk for anxiety disorders through screening in schools: a cluster randomised controlled trial**

Supporting Information

Table of Contents

[Appendix S1: Measure of social, emotional and mental health and wellbeing provision in schools 2](#_Toc213915756)

[Appendix S2: Summary of criteria used to assess school social, emotional, mental health and wellbeing provision 3](#_Toc213915757)

[Appendix S3: Minor adaptations to content of OSI for the MYCATS Trial 6](#_Toc213915758)

[Appendix S4: Secondary clinical outcomes 7](#_Toc213915759)

[Table S1: Number (%) of children screened and enrolled on the trial according to school-level deprivation 10](#_Toc213915760)

[Table S2: Summary of characteristics for children eligible for the trial according to enrolment status 11](#_Toc213915761)

[Table S3: Completion of OSI online modules and support calls in the Intervention arm (N=434) 13](#_Toc213915762)

[Table S4: Therapist and supervisor time (minutes) spent on intervention and supervision for OSI users 14](#_Toc213915763)

[Table S5: Provision of follow-up data by trial arm status 15](#_Toc213915764)

[Table S6: Summary of demographic characteristics by trial arm status and provision primary outcome data 16](#_Toc213915765)

[Table S7: Summary (mean (SD)) of outcomes at baseline by trial arm status and provision of primary outcome data 22](#_Toc213915766)

[Table S8: Summary of outcomes at 6-weeks 24](#_Toc213915767)

[Table S9: Analysis of secondary outcomes at 12-weeks based on complete case analysis 25](#_Toc213915768)

[Table S10: Analysis of outcomes at 12-months based on complete case analysis 26](#_Toc213915769)

[Table S11: Summary of responses to closed questions on the baseline acceptability questionnaire 28](#_Toc213915770)

[Table S12: Summary of participant feedback provided in free text responses on baseline acceptability questionnaire 29](#_Toc213915771)

[Table S13: Summary of responses to closed questions on the 12-week acceptability questionnaire 31](#_Toc213915772)

[Table S14: Summary participant feedback provided in free text responses on 12-week acceptability questionnaire (intervention arm, N=342) 34](#_Toc213915773)

[Table S15: Summary participant feedback provided in free text responses on 12-week acceptability questionnaire (Usual school practice arm, N=355) 36](#_Toc213915774)

[Table S16: Summary of responses to closed questions on the 12-month acceptability questionnaire 38](#_Toc213915775)

[Table S17: Summary participant feedback provided in free text responses on 12-month acceptability questionnaire (intervention arm, N=301) 41](#_Toc213915776)

[Table S18: Summary participant feedback provided in free text responses on 12-month acceptability questionnaire (Usual school practice arm, N=308) 43](#_Toc213915777)

[Table S19: Reported Adverse Events 44](#_Toc213915778)

# Appendix S1: Measure of social, emotional and mental health and wellbeing provision in schools

A nominated staff member was asked to complete a bespoke questionnaire to assess the school’s social, emotional and mental health and wellbeing provision for pupils in Reception to Year 2, including the following open questions:

- What does your school do to promote positive social and emotional health and wellbeing among its pupils in reception to Year 2? Please describe any activities that your school has in place.
- Does your school attempt to identify Reception-Year 2 pupils who might be at-risk of developing social and emotional health difficulties? If yes, how does your school offer support for such pupils? Please describe any specific interventions that your school has in place.
- Does your school attempt to identify Reception-Year 2 pupils with particular social and emotional health needs? If yes, how does your school offer support for such pupils? Please describe any specific interventions that your school has in place.
- If your school works with external services to support Reception-Year 2 pupils' social and emotional health and wellbeing, please give details.
- Please describe any support that your school offers to the families of those Reception-Year 2 pupils with identified social and emotional health needs.
- For those pupils with identified social and emotional health needs, how is their progress assessed? Please list any tools/measures that are used to assess social and emotional health status, e.g. Strengths and Difficulties Questionnaire.
- What plans and policies are in place at your school to support social and emotional health and wellbeing provision?
- What does your school think is the key to success in supporting pupils' social and emotional health and wellbeing?

Responses to these open questions were assessed against criteria in ten areas detailed in Appendix 2. Each area was scored from 0 to 3 (0=no evidence of criteria, 1= evidence of some criteria, 2= evidence of most criteria, 3= evidence of all/almost all criteria) and summed to provide a total score (possible range 0 to 30). The criteria and scoring were developed using both statutory and recommended guidance for schools in England (top-down approach) and collected responses from schools (bottom-up approach). The process to develop the criteria and scoring, and school responses will be reported in detailed elsewhere.

# Appendix S2: Summary of criteria used to assess school social, emotional, mental health and wellbeing provision

|  | **Summary of Criteria** |
| --- | --- |
| 1. Whole school culture |  |
| 1a. Environment, ethos, values | - School environment is a structured, safe, calm, supportive, caring, and nurturing environment - Established school values and ethos that integrate the whole school approach - Frequent reflection, monitoring and evaluation of the whole school approach on mental health and wellbeing - School leadership fosters ethos/values/environment that integrates the whole school approach on mental health and wellbeing - Collaborative approach to designing/implementing/evaluating the whole school approach on mental health and wellbeing |
| 1b. Curriculum and universal teaching practices | - Established stand-alone, universal PSHE/RSE curriculum. - Curriculum and universal teaching practices aim to normalise, foster openness and reduce stigma, and foster resilience, use as preventative strategy, and take a non-judgmental strengths-based approach - Learning from universal curriculum is embedded into the wider curriculum and reinforced through wider activities within the whole school (the hidden curriculum) - Key messages from universal curriculum are communicated to parents to implement at home - Adaptions are made for the school population and context, including current or culturally relevant issues. - Effective student collaboration and student voice evident in the development and on ongoing basis - Curriculum/universal practices are evidence-based and culturally appropriate. - Learning from universal curriculum is assessed, with validated tools |
| 1c. Policies | - Published, uptodate school policies are available to the school community. - Policy related to pupil wellbeing and safeguarding is addressed in statutory school policy documents (e.g. Accessibility plan, Behaviour in Schools policy) that are available to the school community - A specific Mental Health and Wellbeing policy is available to the school community - Policy related to mental health and wellbeing are collaboratively designed/implemented/evaluated. |
| 2 Identification |  |
| 2a. What to identify and consider | - Identification of children's emotional, behavioural and interpersonal needs - Understanding the difference between response to adversity and mental health difficulties; awareness that some children can internalise their distress so it can be difficult to identify; understanding presentations may differ between the school and home environments. - Identification of risk factors, including awareness and understanding of the cumulative effect and interaction of risk factors - Consider the possibility of comorbidities and/or multiple needs. - Consider availability of support and additional actions that will be needed where there may be delays accessing services - When identifying support needed in the short term, consider monitoring or longer-term support needed. |
| 2b. Methods or processes for identification | - Regular observation, reflection and review - Using professional curiosity - Documenting changes in behaviours and symptoms at school and home. - Using clear guidance, policy, and processes within school - Liaising with other services when appropriate to obtain information related to possible unmet need - Using pre-existing data, e.g. attendance data - Parent reporting. This can be encouraged through open door policies, visible or assigned staff members, building good relationships with parents/carers - Use both parent and child voice - Universal mental health and wellbeing assessments, using validated tools and using available data (e.g. national data) to meaningful interpret responses/scores - Consider adaptations that may be needed e.g. for children with SEN, where English is a second language - Act quickly following identification and avoid assumptions that others have reported concerns. |
| 3. Offering, delivering and evaluating targeted support | - Collaborative approach to selecting and deciding on targeted support and any adjustments needed. This includes involving children and parents in the decision making process. - System for sharing planned and implemented support, and progress with the wider school, with appropriate consent for information sharing in place. - Targeted interventions and support provided, with recommended evidence-based interventions/support prioritised. Where suitable intervention/support with strong evidence-base to support effectiveness is not available, the available evidence is considered to inform decisions on selected intervention/support (e.g. evidence-informed, interventions based on adapted evidence-based approach) - Established links with a range of external support services to support signposting and referrals to external support as needed (e.g. CAMHS, wider community support) - Targeted interventions/support and progress are evaluated, with evaluations used to inform decisions about ongoing/additional support. This includes using individual pupil plans for monitoring/reviewing progress and incorporating parent and child voice in feedback/evaluation. Validated outcome measures are used meaningfully to monitor progress and inform decisions about ongoing/additional support. Monitoring/evaluation includes assessing learning/attainment progress/outcomes. |
| 4. Leadership and Management | - School leadership and management commitment to whole school approach to mental health and wellbeing. - School funds and resources managed appropriately to optimise supporting mental health and wellbeing - School leadership and management ensure policies and procedures are fully understood and followed by all staff - Established system for reviewing, sustaining and monitoring plans and policies in practice through school improvement plans |
| 5. Staff Training | - Regular CDP staff training on children's mental health and wellbeing, including identification of common mental health problems. Staff Training needs continuously reviewed. - Regular Safeguarding training for all staff, including regular updates as needed - Staff training is appropriate for staff role and responsibilities. Some training for all staff (e.g. including lunchtime supervisors) to support whole school approach. Additional training for some roles. - Appropriate supervision and support for staff provided |
| 6. Staff Wellbeing | - Established policy and procedures for promoting supporting staff wellbeing. This includes signposting staff to appropriate external support following the Workplace Wellbeing National Standards. - Staff provided with protected time for supervision and professional development activities - Established system for peer support and supervision |
| 7. Parents, Carers, and Families | - Working with parents/carers and families. Collaborative and supportive approach, and includes parental involvement in targeted support. - Effective communication with all parents/carers about support that is offered and available to all pupils. - Supporting parents/carers to support and protect their child's mental health. This may include offering parent-led programmes and support, and identifying parental mental health needs |

# Appendix S3: Minor adaptations to content of OSI for the MYCATS Trial

| **Adaptation Category** | **Nature of adaptation** |
| --- | --- |
| Clinical language amended | - Amended ‘therapist’ to ‘wellbeing practitioner’ - Amended ‘therapy session’ to ‘support session’ - Amended ‘child anxiety treatment’ to ‘OSI’ or ‘OSI programme’ |
| Routine outcome measures amended | - Removed RCADS/RCADS subscales as may not be relevant for this population and to reduce parent burden |
| Module content amended | - Replaced module videos to ensure content was suitable for this population, including new videos addressing emerging anxiety difficulties and no current anxiety difficulties - Amended ‘problems with anxiety’ to ‘fears and worries’, ‘building confidence’ and ‘preventing anxiety’ - Example scenarios and rewards amended for this population/age group |
| Resources | - Added a downloadable ‘parent anxiety’ resource - Amended list of external resources for this population |

 RCADS=Revised Children’s Anxiety and Depression Scale

# Appendix S4: Secondary clinical outcomes

Risk factors, broader child clinical outcomes and additional intervention targets were assessed at 12-weeks and 12-months post-randomisation using the following parent-report questionnaires.

*Risk factors*

Child anxiety symptoms, behavioural inhibition and parent anxiety were assessed using the same measures that we used at screening.

Child anxiety symptoms were assessed using the **Preschool Anxiety Scale (PAS)** total score (Spence et al., 2001). Parents rate 28 items on a 5-point scale (scored 0-4) and responses summed to provide a total score (possible range 0-112).

The 7-item **Approach subscale of the Short Temperament Scale for Children (STSC-A)** (Sanson et al., 1996) was used to assess behavioural inhibition. Items are rated on a 6-point (scored 1-6, with 4 items reverse scored) and summed to provide the total score (range 7-42).

**Parent anxiety** was measured using the **Generalised Anxiety Disorder Scale (GAD-7)** (Spitzer et al., 2006). Responses to the 7-items (scored 0-3) are summed to provide the total score (range 0-21).

*Broader clinical outcomes*

The 18-item **Child Anxiety Life Interference Scale-Preschool Version (CALIS-PV)** (Gilbertson et al., 2017) was used to assess the impact of the child’s anxiety on their life and their parent’s life. Adapted from a similar questionnaire designed for older children and adolescents, the CALIS-PV has been shown to be a reliable and valid measure of anxiety-related interference in daily interference among young children aged 3-7 years (Gilbertson et al., 2017). Items scores are summed to provide a total score (items scored 0-4; total score range 0-72).

To assess children’s externalising symptoms, we used the parent-report version of the **Strengths and Difficulties-Externalising Scale** (SDQ-E; 10 items scored 0-2; total score range 0–20) (Goodman, 1997). The parent-reported SDQ is designed for children and young people aged 4-17 years and its strong psychometric properties are well established in community and clinical populations (see <https://www.sdqinfo.org> for full details).

*Additional intervention targets*

We assessed additional parent and child behaviours that CBT interventions for child anxiety are designed to target.

The **Parent Overprotection Measure** (POM) (Rapee et al., 2024) was used to assess parent behaviours that may limit a child’s exposure to situations that are perceived as potentially risky, threatening or anxiety-provoking. It has shown good psychometric properties among young children aged 3-6 years. Parents rate 19 items on a 5-point scale (0-4), with item scores summed to provide a total score (range 0-76).

We assessed parenting self-efficacy using the widely used 7-item **self-efficacy subscale of the Parenting Sense of Competence Scale** (PSOC-SE) (Johnston & Mash, 1989; Jones & Prinz, 2005). Items are rated on a 6-point scale and scored 1-6, and summed item scores provide the total score (7-42, with higher scores indicating higher parenting self-efficacy).

Child behavioural avoidance was measured using the **Child Avoidance Measure** (CAMP) (Whiteside et al., 2013) which has shown good psychometric properties in primary-school aged children. It consists of 8-items rated on a 4-point scale (0-3) that are summed to provide a total score (range 0-24).

The 17-item **Responses to Uncertainty and Low Environmental Structure** (RULES) (Sanchez et al., 2017) was used to measure child intolerance of uncertainty. The RULES is designed for children aged 3-10 years, with evidence to support its reliability and validity (Sanchez et al., 2017). Items are rated on a 5-point scale, scored 1-5 and providing a total score from 17-85.

To assess child coping efficacy, we used an adapted version of the parent-report **Coping Questionnaire** (CQ-P) (Crane & Kendall, 2020) that has been used with children as young as 6 years (Lau et al, 2010). On the original measure, anxiety-provoking situations (derived from a preceding diagnostic interview) are specified for the respondent. Instead, at baseline we asked parents to provide three situations where their child feels scared or worried, and they then rated how well their child was able to cope in each of these situations at baseline and each follow-up. As specified in the statistical analysis plan, we used the score (1-7) for the first anxiety-provoking situation in the analysis, with higher scores indicating higher coping efficacy.

**References**

Crane, M. E., & Kendall, P. C. (2020). Psychometric Evaluation of the Child and Parent Versions of the Coping Questionnaire. *Child Psychiatry & Human Development*, *51*(5), 709–720. https://doi.org/10.1007/s10578-020-00975-w

Gilbertson, T. J., Morgan, A. J., Rapee, R. M., Lyneham, H. J., & Bayer, J. K. (2017). Psychometric properties of the child anxiety life interference scale – Preschool version. *Journal of Anxiety Disorders*, *52*, 62–71. https://doi.org/10.1016/j.janxdis.2017.10.002

Goodman, R. (1997). The Strengths and Difficulties Questionnaire: a research note. *Journal of Child Psychology and Psychiatry*, *38*(5), 581–586. https://doi.org/10.1111/j.1469-7610.1997.tb01545.x

Johnston, C., & Mash, E. J. (1989). A Measure of Parenting Satisfaction and Efficacy. *Journal of Clinical Child Psychology*, *18*(2), 167–175. https://doi.org/10.1207/s15374424jccp1802_8

Jones, T., & Prinz, R. (2005). Potential roles of parental self-efficacy in parent and child adjustment: A review. *Clinical Psychology Review*, *25*(3), 341–363.

Rapee, R. M., Edwards, S. L., Mabood, S., & Freeman, J. Y. A. (2024). Psychometric Properties of a Self-Report Measure of Overprotective Parenting: The Parental Overprotection Measure (POM). *Child Psychiatry & Human Development*. https://doi.org/10.1007/s10578-024-01801-3

Sanchez, A. L., Cornacchio, D., Chou, T., Leyfer, O., Coxe, S., Pincus, D., & Comer, J. S. (2017). Development of a scale to evaluate young children’s responses to uncertainty and low environmental structure. *Journal of Anxiety Disorders*, *45*, 17–23. https://doi.org/10.1016/j.janxdis.2016.11.006

Sanson, A., Pedlow, R., Cann, W., Prior, M., & Oberklaid, F. (1996). Shyness ratings: Stability and correlates in early childhood. *International Journal of Behavioral Development*, *19*(4), 705–724.

Spence, S. H., Rapee, R., McDonald, C., & Ingram, M. (2001). The structure of anxiety symptoms among preschoolers. *Behaviour Research and Therapy*, *39*(11), 1293–1316. https://doi.org/10.1016/S0005-7967(00)00098-X

Spitzer, R. L., Kroenke, K., Williams, J. B. W., & Löwe, B. (2006). A brief measure for assessing generalized anxiety disorder: The GAD-7. *Archives of Internal Medicine*. https://doi.org/10.1001/archinte.166.10.1092

Whiteside, S. P. H., Gryczkowski, M., Ale, C. M., Brown-Jacobsen, A. M., & McCarthy, D. M. (2013). Development of child- and parent-report measures of behavioral avoidance related to childhood anxiety disorders. *Behavior Therapy*, *42*(2), 325–337. https://doi.org/10.1016/j.beth.2013.02.006

Lau, W. Y., Chan, C. K. Y., Li, J. C. H., & Au, T. K. F. (2010). Effectiveness of group cognitive-behavioral treatment for childhood anxiety in community clinics. *Behaviour research and therapy*, *48*(11), 1067-1077.

# Table S1: Number (%) of children screened and enrolled on the trial according to school-level deprivation

|  | **Above average proportion of pupils eligible for free school meals**  **(45 schools)** | **Below average proportion of pupils eligible for free school meals**  **(50 schools)** |
| --- | --- | --- |
| Number of children screened, mean (SD), median (IQR) | 18.0 (12.5), 14.0 (8.5-25.5) | 30.3 (18.6), 27.0 (14.5-39.3) |
| Number of children enrolled on the trial, mean (SD), median (IQR) | 6.9 (5.3), 8.0 (5.0-13.0) | 11.1 (6.6), 13.0 (8.0-20.0) |

# Table S2: Summary of characteristics for children eligible for the trial according to enrolment status

|  | **Eligible children who did not enrol (N=307)** | **Enrolled children (N=865)** |
| --- | --- | --- |
| Child year group |  |  |
| Reception, 4-5 years | 94 (31%) | 254 (29%) |
| Year 1, 5-6 years | 87 (28%) | 297 (34%) |
| Year 2, 6-7 years | 126 (41%) | 314 (36%) |
| Child gender |  |  |
| Female | 154/305 (50%) | 432 (50%) |
| Male | 151/305 (50%) | 433 (50%) |
| Child ethnicity |  |  |
| Asian^††^ | 19/305 (6%) | 28/864 (3%) |
| Black^⸸^ | 5/305 (2%) | 9/864 (1%) |
| Mixed^¥^ | 18/305 (6%) | 69/864 (8%) |
| White^‡^ | 254/305 (83%) | 751/864 (87%) |
| Other^#^ | 6/305 (2%) | 4/864 (<1%) |
| Prefer not to say | 3/305 (<1%) | 3/864 (<1%) |
| Parent gender |  |  |
| Female | 289/305 (95%) | 810 (94%) |
| Male | 15/305 (5%) | 54 (6%) |
| Other / prefer not to say | 1/305 (<1%) | 1 (<1%) |
| Parent ethnicity |  |  |
| Asian^††^ | 19/303 (6%) | 33/858 (4%) |
| Black^⸸^ | 6/303 (2%) | 12/858 (1%) |
| Mixed^¥^ | 7/303 (2%) | 25/858 (3%) |
| White^‡^ | 260/303 (86%) | 775/858 (90%) |
| Other^#^ | 8/303 (3%) | 9/858 (1%) |
| Prefer not to say | 3/303 (1%) | 4/858 (<1%) |
| Parent is partnered | 220/305 (72%) | 714/865 (83%) |
| Type of housing |  |  |
| Privately rented | 82/299 (27%) | 152/858 (18%) |
| Council or housing association rented | 65/299 (22%) | 134/858 (16%) |
| Mortgage/owned | 138/299 (46%) | 560/858 (65%) |
| Other | 8/299 (3%) | 8/858 (1%) |
| Prefer not to say | 6/299 (2%) | 4/858 (<1%) |
| Parent education |  |  |
| School completion | 41/304 (13%) | 52 (6%) |
| Further education | 124/304 (41%) | 293 (34%) |
| Higher education | 74/304 (24%) | 273 (32%) |
| Postgraduate education | 32/304 (11%) | 226 (26%) |
| None of the above | 14/304 (5%) | 9 (1%) |
| Prefer not to say | 19/304 (6%) | 12 (1%) |
| Parent employment |  |  |
| Unemployed | 46/301 (15%) | 62/863 (7%) |
| Part-time | 93/301 (31%) | 339/863 (39%) |
| Full time | 91/301 (30%) | 289/863 (33%) |
| Student | 12/301 (4%) | 19/863 (2%) |
| Retired | 2/301 (<1%) | 1/863 (<1%) |
| Homemaker | 34/301 (11%) | 105/863 (12%) |
| Other | 15/301 (5%) | 38/863 (4%) |
| Prefer not to say | 8/301 (3%) | 10/863 (1%) |
| Screening outcome |  |  |
| Screened positive for child anxiety | 199 (65%) | 634 (73%) |
| Screened positive for behavioural inhibition | 105 (34%) | 258 (30%) |
| Screened positive for parent anxiety | 185 (61%) | 477 (55%) |

Data are n (%) or n/N (%).

††Includes Asian or Asian British Indian, Pakistani, any other Asian background

⸸Includes Black or Black British African, Caribbean, any other Black background

¥Includes White and Black Caribbean, White and Black African, White and Asian, any other mixed background

‡Includes British, Irish, any other White background

#Includes Chinese

# Table S3: Completion of OSI online modules and support calls in the Intervention arm (N=434)

|  | **Online module complete** | **Support call complete** |
| --- | --- | --- |
| Module 0 | 394/434 (90.8%) | 386/434 (88.9%) |
| Module 1 | 378/434 (87.1%) | 374/434 (86.2%) |
| Module 2 | 362/434 (83.4%) | 357/434 (82.3%) |
| Module 3 | 345/434 (79.5%) | 341/434 (78.6%) |
| Module 4 | 332/434 (76.5%) | 332/434 (76.5%) |
| Module 5 | 328/434 (75.6%) | 327/434 (75.3%) |
| Module 6 | 317/434 (73.0%) | 319/434 (73.5%) |
| Module 7 (follow-up)* | 271/434 (62.4%) | 308/434 (71.0%) |

Data are n/N (%). *Module 7 online module does not include new intervention content, only routine outcome measures

# Table S4: Therapist and supervisor time (minutes) spent on intervention and supervision for OSI users

|  | N | Mean (SD) | Median (IQR) |
| --- | --- | --- | --- |
| **Intervention related time** | **407** | **306.06 (127.09)** | **330 (141)** |
| Parent support sessions | 407 | 152.33 (69.68) | 165 (76) |
| Preparation | 407 | 56.63 (24.63) | 60.65 (35) |
| Admin and additional contact | 407 | 97.10 (49.41) | 95.00 (58.15) |
| **Supervision related time** | **380** | **78.00 (75.11)** | **61.64 (67**.**93)** |
| Supervision session by therapists | 380 | 30.64 (26.89) | 25.00 (25.5) |
| Supervision session by supervisors | 380 | 30.64 (26.89) | 25.00 (25.5) |
| Other time by therapists | 380 | 11.09 (14.09) | 7.23 (9.78) |
| Other time by supervisors | 380 | 5.61 (11.82) | 1.67 (4.46) |

Note: This table summarises the time (minutes) spent on a participant in the intervention arm. This calculation is based on the participants who are recorded at least once in the treatment and supervision logs.

# Table S5: Provision of follow-up data by trial arm status

| **Assessment** | **Intervention** | **Usual school practice** | **Overall** |
| --- | --- | --- | --- |
|  | **N = 434** | **N = 431** | **N = 865** |
|  |  |  |  |
| Baseline, n (%) | 434 (100%) | 431 (100%) | 865 (100%) |
| 6 weeks, n (%) | 362 (83.4%) | 384 (89.1%) | 746 (86.2%) |
| 12 weeks, n (%) | 350 (80.6%) | 361 (83.8%) | 711 (82.2%) |
| 12 months (any outcome), n (%) | 335 (77.2%) | 342 (79.4%) | 677 (78.3%) |
| 12 months (diagnostic assessment), n (%) | 310 (71.4%) | 312 (72.4%) | 622 (71.9%) |
|  |  |  |  |

# Table S6: Summary of demographic characteristics by trial arm status and provision primary outcome data

|  | **Intervention** | |  | **Usual School Practice** | |
| --- | --- | --- | --- | --- | --- |
|  | **Primary outcome data (N = 310)^a^** | **No primary outcome data (N = 124)^b^** |  | **Primary outcome data (N = 312)^c^** | **No primary outcome data (N = 119)^d^** |
| **Child characteristics** | | | | | |
| Cohort |  |  |  |  |  |
| Cohort 1, % | 10.0 | 16.1 |  | 12.8 | 15.1 |
| Cohort 2, % | 29.0 | 29.0 |  | 30.8 | 24.4 |
| Cohort 3, % | 28.1 | 25.0 |  | 26.6 | 29.4 |
| Cohort 4, % | 22.6 | 23.4 |  | 27.9 | 26.1 |
| Cohort 5, % | 10.3 | 6.5 |  | 1.9 | 5.0 |
| Child year group |  |  |  |  |  |
| Reception, % | 28.1 | 29.8 |  | 28.5 | 34.5 |
| Year 1, % | 35.2 | 34.7 |  | 36.5 | 26.1 |
| Year 2, % | 36.8 | 35.5 |  | 34.9 | 39.5 |
| Child age, mean (SD) | 6.3 (0.9) | 6.2 (0.9) |  | 6.2 (0.9) | 6.2 (0.9) |
| Child gender |  |  |  |  |  |
| Male, % | 48.7 | 51.6 |  | 50.6 | 50.4 |
| Female, % | 51.3 | 48.4 |  | 49.4 | 49.6 |
| Fostering/adoption |  |  |  |  |  |
| No, % | 99.0 | 97.6 |  | 96.8 | 98.3 |
| Fostered, % | 0 | 0.8 |  | 0.6 | 0 |
| Adopted, % | 0.7 | 0.8 |  | 2.3 | 0.8 |
| Prefer not to say, % | 0.3 | 0.8 |  | 0.3 | 0.8 |
| Child ethnicity |  |  |  |  |  |
| White, % | 88.0 | 81.5 |  | 87.8 | 89.7 |
| Black, % | 0.6 | 1.6 |  | 1.3 | 0.9 |
| Mixed, % | 7.1 | 10.5 |  | 8.3 | 6.9 |
| Asian, % | 3.2 | 5.6 |  | 2.6 | 2.6 |
| Other, % | 1.0 | 0.8 |  | 0 | 0 |
| **Parent characteristics** | | | | | |
| Parent age, mean (SD) | 37.4 (5.1) | 35.1 (6.3) |  | 37.6 (5.3) | 35.6 (5.3) |
| Parent gender |  |  |  |  |  |
| Male, % | 7.4 | 1.6 |  | 7.1 | 5.9 |
| Female, % | 92.6 | 98.4 |  | 92.6 | 94.1 |
| Other, % | 0 | 0 |  | 0.3 | 0 |
| Relationship to child |  |  |  |  |  |
| Mother, % | 92.3 | 97.6 |  | 91.3 | 93.3 |
| Father, % | 7.4 | 1.6 |  | 7.1 | 5.9 |
| Other, % | 0.3 | 0.8 |  | 1.6 | 0.8 |
| Parent ethnicity |  |  |  |  |  |
| White, % | 90.9 | 86.0 |  | 91.6 | 93.1 |
| Black, % | 1.0 | 2.5 |  | 1.6 | 0.9 |
| Mixed, % | 3.3 | 3.3 |  | 2.3 | 3.4 |
| Asian, % | 3.6 | 6.6 |  | 3.5 | 2.6 |
| Other, % | 1.3 | 1.7 |  | 1.0 | 0 |
| Number of children in household |  |  |  |  |  |
| None, % | 0 | 0 |  | 0.3 | 0 |
| One, % | 20.7 | 23.4 |  | 22.6 | 25.2 |
| Two, % | 58.6 | 51.6 |  | 55.8 | 47.9 |
| Three, % | 16.8 | 16.1 |  | 16.1 | 16.8 |
| Four, % | 2.9 | 4.8 |  | 3.2 | 5.9 |
| Five, % | 0.3 | 2.4 |  | 1.6 | 3.4 |
| Six, % | 0.6 | 1.6 |  | 0.3 | 0.8 |
| Type of housing |  |  |  |  |  |
| Privately rented, % | 15.3 | 19.4 |  | 17.5 | 22.7 |
| Council rented, % | 7.5 | 16.1 |  | 5.8 | 12.6 |
| Housing association, % | 5.5 | 9.7 |  | 5.8 | 9.2 |
| Mortgage/owned, % | 71.3 | 51.6 |  | 69.8 | 52.1 |
| Other, % | 0.3 | 0.8 |  | 0.6 | 3.4 |
| Prefer not to say, % | 0 | 2.4 |  | 0.3 | 0 |
| Partner |  |  |  |  |  |
| Yes, % | 87.1 | 73.4 |  | 86.5 | 69.7 |
| No, % | 12.3 | 23.4 |  | 12.8 | 27.7 |
| Prefer not to say, % | 0.6 | 3.2 |  | 0.6 | 2.5 |
| Cohabit with partner |  |  |  |  |  |
| Yes, % | 96.7 | 93.4 |  | 98.1 | 94.0 |
| No, % | 3.3 | 4.4 |  | 1.9 | 3.6 |
| Prefer not to say, % | 0 | 2.2 |  | 0 | 2.4 |
| Highest level of education |  |  |  |  |  |
| School completion, % | 4.5 | 6.5 |  | 5.1 | 11.8 |
| Further education, % | 30.6 | 42.7 |  | 32.1 | 37.8 |
| Higher education, % | 32.9 | 29.8 |  | 34.9 | 21.0 |
| Postgraduate qualification, % | 30.0 | 16.1 |  | 26.6 | 25.2 |
| None of the above, % | 1.3 | 0.8 |  | 0.3 | 2.5 |
| Prefer not to say, % | 0.6 | 4.0 |  | 1.0 | 1.7 |
| Partner’s education |  |  |  |  |  |
| School completion, % | 14.5 | 20.2 |  | 12.3 | 15.9 |
| Further education, % | 31.2 | 38.2 |  | 38.7 | 47.6 |
| Higher education, % | 23.4 | 13.5 |  | 25.7 | 17.1 |
| Postgraduate qualification, % | 26.4 | 18.0 |  | 21.2 | 14.6 |
| None of the above, % | 3.0 | 4.5 |  | 1.1 | 3.7 |
| Prefer not to say, % | 1.5 | 5.6 |  | 1.1 | 1.2 |
| Employment |  |  |  |  |  |
| Unemployed, % | 5.5 | 14.5 |  | 4.5 | 10.9 |
| Part-time, % | 41.2 | 31.5 |  | 42.3 | 34.5 |
| Full-time, % | 34.1 | 32.3 |  | 33.3 | 33.6 |
| Sheltered/supported employment, % | 0 | 0 |  | 0 | 0 |
| Student, % | 2.9 | 2.4 |  | 1.9 | 0.8 |
| Retired, % | 0 | 0 |  | 0 | 0.8 |
| Homemaker, % | 12.0 | 13.7 |  | 11.2 | 13.4 |
| Other, % | 3.9 | 3.2 |  | 5.8 | 3.4 |
| Prefer no to say, % | 0.3 | 2.4 |  | 1.0 | 2.5 |
| Partner’s employment |  |  |  |  |  |
| Unemployed, % | 3.4 | 3.3 |  | 1.9 | 3.6 |
| Part-time, % | 5.6 | 3.3 |  | 4.5 | 8.4 |
| Full-time, % | 86.9 | 84.6 |  | 85.9 | 79.5 |
| Sheltered/supported employment, % | 0 | 0 |  | 0 | 0 |
| Student, % | 0 | 0 |  | 1.1 | 0 |
| Retired, % | 0 | 0 |  | 0.4 | 0 |
| Homemaker, % | 0.7 | 1.1 |  | 2.6 | 2.4 |
| Other, % | 2.6 | 3.3 |  | 3.7 | 4.8 |
| Prefer no to say, % | 0.7 | 4.4 |  | 0 | 1.2 |
| Household income |  |  |  |  |  |
| <£16,000, % | 11.7 | 22.7 |  | 9.7 | 25.0 |
| £16,001 to £30,000, % | 12.7 | 10.1 |  | 12.3 | 15.5 |
| £30,001 to £40,000, % | 9.3 | 8.4 |  | 10.7 | 11.2 |
| £40,001 to £50,000, % | 10.7 | 10.1 |  | 11.0 | 8.6 |
| £50,001 to £60,000, % | 12.3 | 8.4 |  | 13.3 | 6.9 |
| £60,001 to £70,000, % | 9.3 | 11.8 |  | 8.1 | 3.4 |
| £70,001 to £80,000, % | 9.0 | 1.7 |  | 5.5 | 9.5 |
| £80,001 to £90,000, % | 5.7 | 2.5 |  | 7.1 | 6.0 |
| £90,001 to £120,000, % | 6.3 | 2.5 |  | 7.1 | 3.4 |
| >£120,000, % | 5.3 | 4.2 |  | 4.5 | 4.3 |
| Prefer not to say, % | 7.7 | 17.6 |  | 10.7 | 6.0 |
| Index of Multiple Deprivation decile-based category |  |  |  |  |  |
| 1^st^ (most deprived) , % | 8.7 | 12.1 |  | 7.7 | 15.1 |
| 2^nd^, % | 8.1 | 12.1 |  | 4.8 | 5.0 |
| 3^rd^, % | 14.2 | 14.5 |  | 8.7 | 16.8 |
| 4^th^, % | 7.7 | 12.1 |  | 7.7 | 4.2 |
| 5^th^, % | 10.0 | 6.5 |  | 17.3 | 14.3 |
| 6^th^, % | 11.3 | 12.9 |  | 10.9 | 7.6 |
| 7^th^, % | 9.7 | 4.8 |  | 11.5 | 10.9 |
| 8^th^, % | 12.9 | 8.1 |  | 7.4 | 6.7 |
| 9^th^, % | 10.6 | 8.9 |  | 15.7 | 9.2 |
| 10^th^ (least deprived) , % | 6.8 | 8.1 |  | 8.3 | 10.1 |

^a^ Sample size ranges from 267 to 310

^b^ Sample size ranges from 89 to 124

^c^ Sample size ranges from 269 to 312

^d^ Sample size ranges from 82 to 119

# Table S7: Summary (mean (SD)) of outcomes at baseline by trial arm status and provision of primary outcome data

| **Measure** | **Intervention** | |  | **Usual School Practice** | |
| --- | --- | --- | --- | --- | --- |
|  | **Primary outcome data (N = 310)^a^** | **No primary outcome data (N = 124)^b^** |  | **Primary outcome data (N = 312)^c^** | **No primary outcome data (N = 119)^d^** |
| PAS | 40.8 (16.9) | 39.8 (17.9) |  | 41.1 (16.6) | 46.4 (15.1) |
| STSC Approach Subscale | 24.1 (7.6) | 23.1 (7.6) |  | 24.0 (7.6) | 26.2 (7.9) |
| GAD-7 | 7.2 (4.7) | 8.7 (5.4) |  | 7.1 (4.8) | 8.8 (5.6) |
| CALIS-PV | 21.8 (13.3) | 22.8 (13.2) |  | 22.7 (13.4) | 25.7 (13.5) |
| SDQ-E | 7.9 (4.2) | 8.7 (4.2) |  | 8.6 (4.4) | 9.0 (4.5) |
| POM | 34.3 (11.8) | 37.9 (12.8) |  | 35.4 (12.0) | 36.8 (12.6) |
| PSCS Self-efficacy subscale | 27.6 (5.7) | 28.6 (5.6) |  | 27.4 (5.8) | 28.2 (5.7) |
| RULES | 42.0 (14.5) | 42.7 (15.0) |  | 41.6 (13.4) | 45.3 (13.4) |
| CQ-P | 3.3 (1.4) | 3.1 (1.4) |  | 3.2 (1.5) | 3.1 (1.3) |
| CAMP | 10.7 (5.6) | 10.8 (5.3) |  | 10.6 (5.6) | 11.2 (5.6) |

^a^ Sample size ranges from 305 to 301

^b^ Sample size ranges from 118 to 124

^c^ Sample size ranges from 302 to 312

^d^ Sample size ranges from 115 to 119

For all outcomes higher scores indicate worse functioning, with the exception of PSCS Self-efficacy and CQ-P where higher scores indicate better functioning.

PAS: Pre-school Anxiety Scale; STSC-Approach: Approach subscale of the Short Temperament Scale for Children; GAD-7: Generalised Anxiety Disorder Scale-7; CALIS-PV: Child Anxiety Life Interference Scale-Preschool version; SDQ-E: Strengths and Difficulties Questionnaire Externalising Scale; POM: Parent Overprotection Measure; PSCS Self-efficacy: Self-Efficacy Subscale of the Parenting Sense of Competence Scale; RULES: Responses to Uncertainty and Low Environmental Structure; CQ-P: Coping Questionnaire; CAMP: Child Avoidance Measure

# Table S8: Summary of outcomes at 6-weeks

| **Measure** | **Intervention** | | **Usual School Practice** | | **ICC^a^** |
| --- | --- | --- | --- | --- | --- |
|  | **N** | **mean (SD)** | **N** | **mean (SD)** |  |
| PAS | 362 | 35.9 (17.9) | 384 | 40.0 (17.0) | 0.037 |
| STSC Approach Subscale | 359 | 22.8 (7.6) | 382 | 23.9 (7.5) | 0.035 |
| GAD-7 | 357 | 6.6 (5.1) | 380 | 7.4 (5.2) | 0.043 |
| POM | 355 | 30.6 (12.8) | 379 | 34.6 (12.0) | 0.056 |
| PSCS Self-efficacy subscale | 355 | 29.5 (5.1) | 379 | 28.5 (5.5) | 0.009 |
| RULES | 357 | 42.9 (15.1) | 381 | 44.9 (14.0) | 0.011 |
| CQ-P | 350 | 3.8 (1.4) | 373 | 3.5 (1.5) | 0.023 |
| CAMP | 357 | 11.3 (5.8) | 381 | 11.7 (5.8) | 0.029 |

^a^ intra-cluster (intra-school) correlation coefficient adjusted for trial arm status

For all outcomes higher scores indicate worse functioning, with the exception of PSCS Self-efficacy and CQ-P where higher scores indicate better functioning.

PAS: Pre-school Anxiety Scale; STSC-Approach: Approach subscale of the Short Temperament Scale for Children; GAD-7: Generalised Anxiety Disorder Scale-7; CALIS-PV: Child Anxiety Life Interference Scale-Preschool version; SDQ-E: Strengths and Difficulties Questionnaire Externalising Scale; POM: Parent Overprotection Measure; PSCS Self-efficacy: Self-Efficacy Subscale of the Parenting Sense of Competence Scale; RULES: Responses to Uncertainty and Low Environmental Structure; CQ-P: Coping Questionnaire; CAMP: Child Avoidance Measure

# Table S9: Analysis of secondary outcomes at 12-weeks based on complete case analysis

|  | **Intervention** | | **Usual School Practice** | | **Unadjusted** | | **Adjusted** | | |
| --- | --- | --- | --- | --- | --- | --- | --- | --- | --- |
|  | **N** | **mean (SD)** | **N** | **mean (SD)** | **mean difference** | **ICC^a^** | **mean difference (95% CI)** | **p value** | **ICC^a^** |
| PAS | 350 | 28.8 (17.5) | 361 | 38.3 (17.1) | -9.5 | 0.004 | -8.4 (-10.1 to -6.6) | <0.0001 | 0 |
| STSC Approach Subscale | 350 | 20.9 (7.4) | 361 | 23.6 (7.7) | -2.8 | 0.048 | -2.3 (-3.0 to -1.6) | <0.0001 | 0.014 |
| GAD-7 | 346 | 5.5 (4.8) | 360 | 7.2 (5.6) | -1.7 | 0.042 | -1.8 (-2.4 to -1.2) | <0.0001 | 0 |
| CALIS-PV | 349 | 16.7 (13.0) | 360 | 21.7 (15.2) | -5.1 | 0.020 | -4.3 (-5.6 to -2.8) | <0.0001 | 0 |
| SDQ-E | 349 | 6.9 (4.0) | 360 | 8.1 (4.4) | -1.2 | 0.004 | -0.8 (-1.2 to -0.4) | <0.0001 | 0.013 |
| POM | 342 | 26.2 (12.5) | 355 | 33.4 (12.3) | -7.4 | 0.033 | -6.1 (-7.4 to -4.9) | <0.0001 | 0.008 |
| PSCS Self-efficacy subscale | 342 | 30.4 (5.6) | 355 | 28.6 (5.9) | 1.8 | 0.015 | 1.9 (1.2 to 2.5) | <0.0001 | 0 |
| RULES | 342 | 36.9 (14.6) | 357 | 41.1 (14.4) | -4.2 | 0.029 | -4.1 (-5.5 to -2.7) | <0.0001 | 0 |
| CQ-P | 338 | 4.5 (1.5) | 349 | 3.7 (1.5) | 0.8 | 0 | 0.7 (0.5 to 1.0) | <0.0001 | 0 |
| CAMP | 343 | 9.0 (5.5) | 357 | 10.6 (6.0) | -1.6 | 0 | -1.7 (-2.4 to -1.0) | <0.0001 | 0 |

**^a^**ICC: Intra-cluster (intra-school) correlation coefficient

For all outcomes higher scores indicate worse functioning, with the exception of PSCS Self-efficacy and CQ-P where higher scores indicate better functioning.

PAS: Pre-school Anxiety Scale; STSC-Approach: Approach subscale of the Short Temperament Scale for Children; GAD-7: Generalised Anxiety Disorder Scale-7; CALIS-PV: Child Anxiety Life Interference Scale-Preschool version; SDQ-E: Strengths and Difficulties Questionnaire Externalising Scale; POM: Parent Overprotection Measure; PSCS Self-efficacy: Self-Efficacy Subscale of the Parenting Sense of Competence Scale; RULES: Responses to Uncertainty and Low Environmental Structure; CQ-P: Coping Questionnaire; CAMP: Child Avoidance Measure

# Table S10: Analysis of outcomes at 12-months based on complete case analysis

| **Outcome** | **Intervention** | | **Usual School Practice** | | **Unadjusted** | | **Adjusted** | | |
| --- | --- | --- | --- | --- | --- | --- | --- | --- | --- |
|  | **N** | **mean (SD) /**  **n (%)** | **N** | **mean (SD) / n (%)** | **odds ratio / mean difference** | **ICC^a^** | **odds ratio/ mean difference (95% CI)** | **p-value** | **ICC^a^** |
| **Anxiety disorder diagnosis** | 310 | 21 (6.8%) | 312 | 36 (11.5%) | 0.61 | 0.034 | 0.57 (0.34 to 0.96) | 0.034 | -0.009 |
|  |  |  |  |  |  |  |  |  |  |
| PAS | 314 | 26.4 (16.9) | 321 | 33.3 (17.1) | -6.8 | 0.013 | -6.8 (-8.9 to -4.7) | <0.0001 | 0 |
| STSC Approach Subscale | 313 | 20.1 (7.2) | 320 | 21.6 (7.7) | -1.4 | 0.031 | -1.6 (-2.4 to -0.8) | 0.0002 | 0.004 |
| GAD-7 | 307 | 5.2 (4.4) | 313 | 6.5 (5.1) | -1.4 | 0.006 | -1.5 (-2.0 to -0.9) | <0.0001 | 0 |
| CALIS-PV | 313 | 15.5 (13.8) | 319 | 19.7 (15.7) | -4.2 | 0.022 | -4.0 (-5.8 to -2.2) | <0.0001 | 0 |
| SDQ-E | 311 | 6.6 (4.3) | 317 | 8.1 (4.9) | -1.5 | 0 | -1.0 (-1.5 to -0.6) | <0.0001 | 0.002 |
| POM | 301 | 24.9 (11.5) | 308 | 30.7 (11.8) | -5.9 | 0.019 | -5.1 (-6.5 to -3.8) | <0.0001 | 0 |
| PSCS Self-efficacy subscale | 301 | 30.4 (5.4) | 308 | 29.0 (5.9) | 1.4 | 0 | 1.6 (0.9 to 2.3) | <0.0001 | 0.007 |
| RULES | 301 | 36.1 (15.2) | 308 | 39.9 (15.4) | -3.8 | 0 | -4.5 (-6.3 to -2.7) | <0.0001 | 0 |
| CQ-P | 297 | 4.9 (1.6) | 301 | 4.2 (1.6) | 0.7 | 0.017 | 0.7 (0.4 to 1.0) | <0.0001 | 0.020 |
| CAMP | 301 | 8.0 (5.8) | 308 | 9.9 (6.0) | -1.9 | 0.017 | -2.1 (-3.1 to -1.2) | <0.0001 | 0.029 |

^a^ ICC: Intra-cluster (intra-school) correlation coefficient

For all outcomes higher scores indicate worse functioning, with the exception of PSCS Self-efficacy and CQ-P where higher scores indicate better functioning.

PAS: Pre-school Anxiety Scale; STSC-Approach: Approach subscale of the Short Temperament Scale for Children; GAD-7: Generalised Anxiety Disorder Scale-7; CALIS-PV: Child Anxiety Life Interference Scale-Preschool version; SDQ-E: Strengths and Difficulties Questionnaire Externalising Scale; POM: Parent Overprotection Measure; PSCS Self-efficacy: Self-Efficacy Subscale of the Parenting Sense of Competence Scale; RULES: Responses to Uncertainty and Low Environmental Structure; CQ-P: Coping Questionnaire; CAMP: Child Avoidance Measure

# Table S11: Summary of responses to closed questions on the baseline acceptability questionnaire

| **Item** | **Response / mean (SD)** | **Intervention**  **N=434** | **Usual school practice**  **N=431** | **Total**  **N=865** |
| --- | --- | --- | --- | --- |
| I was happy to complete the questionnaires | Completely disagree | 1/427 (<1%) | 2/424 (<1%) | 3/851 (<1%) |
|  | Disagree | 1/427 (<1%) | 2/424 (<1%) | 3/851 (<1%) |
|  | Neither agree nor disagree | 17/427 (4%) | 15/424 (4%) | 32/851 (4%) |
|  | Agree | 171/427 (40%) | 192/424 (45%) | 363/851 (43%) |
|  | Completely agree | 237/427 (56%) | 213/424 (50%) | 450/851 (53%) |
|  | Mean (SD), n | 4.50 (0.61), n=427 | 4.44 (0.64), n=424 | 4.47 (0.62), n=851 |
| The feedback I received about the questionnaire responses was clear and easy to understand | Completely disagree | 1/428 (<1%) | 1/420 (<1%) | 2/848 (<1%) |
|  | Disagree | 7/428 (2%) | 6/420 (1%) | 13/848 (2%) |
|  | Neither agree nor disagree | 54/428 (13%) | 58/420 (14%) | 112/848 (13%) |
|  | Agree | 180/428 (42%) | 202/420 (48%) | 382/848 (45%) |
|  | Completely agree | 186/428 (43%) | 153/420 (36%) | 339/848 (40%) |
|  | Mean (SD), n | 4.27 (0.76), n=428 | 4.19 (0.74), n=420 | 4.23 (0.75), n=848 |
| I was happy with how I received feedback about the questionnaire responses and my eligibility for the trial | Completely disagree | 0/428 (0%) | 1/420 (<1%) | 1/848 (<1%) |
|  | Disagree | 4/428 (<1%) | 2/420 (<1%) | 6/848 (<1%) |
|  | Neither agree nor disagree | 37/428 (9%) | 42/420 (10%) | 79/848 (9%) |
|  | Agree | 189/428 (44%) | 209/420 (50%) | 398/848 (47%) |
|  | Completely agree | 198/428 (46%) | 166/420 (40%) | 364/848 (43%) |
|  | Mean (SD), n | 4.36 (0.68), n=428 | 4.28 (0.68), n=420 | 4.32 (0.68), n=848 |

Data were not available for all participants. Data are n/N (%), unless otherwise stated

# Table S12: Summary of participant feedback provided in free text responses on baseline acceptability questionnaire

| **Feedback category** | **Frequency n/N (%)** | **Exemplar quotes** |
| --- | --- | --- |
| Positive feedback | 39/851 (5%) | I am really excited to be part of this movement |
|  |  | The staff on the end of the phone have been really friendly and understanding. Been great with explaining, and if needing help. |
|  |  | The lovely lady from the study was so kind and listened to me. I really appreciated her time she on the phone with me. |
|  |  | I am looking forward to taking on board any new ideas or ways of thinkings to benefit my child and family and prevention is better than cure. |
| Discomfort/distress answering questions | 3/851 (<1%) | Answering the questions made me feel uncomfortable as I probably came across as I do not protect my children. |
|  |  | I found myself particularly uncomfortable around the questionnaire that assessed my views of my parenting as there were questions that were twofold and I ended up choosing answers that weren't strictly representative of my true view, I think we are invited to be either super confident or somewhat self depreciating. |
|  |  | Some of the questions were triggering and made me feel uncomfortable. Doing the second set (baseline) questionnaires with a staff member was helpful and easier to understand. |
| Negative feedback about length/relevance/clarity of some questions or response formats | 42/851 (5%) | Some of the questions are hard to answer with the multiple choice answers (the double negative ones) due to the wording. |
|  |  | Some of the questions were tricky for me to answer as my child is different at home from when she is at school. |
|  |  | One question referred to siblings but [my child] does not have any siblings. |
|  |  | Hard to answer on behalf of my child for many of the questions. |
|  |  | A lot of the questions require more nuance than the yes/no options allowed (and the most applicable choice may give the wrong impression. |
|  |  | There's a LOT of questions. |
| Negative experience of the study procedures, outside completing questionnaires | 8/851 (<1%) | Only issue I had was technical, in downloading the PDF diary, and in the surveys themselves having a couple of issues, but other than that all fine. |
|  |  | Couldn't access [feedback/eligibility letter] due to code sending issues . |
|  |  | Too many people called me. |
| Suggested change to study procedures | 8/851 (<1%) | We feel [our child] would benefit more from face to face in school rather than online tools as he will engage more in the process. |
|  |  | I was very happy with our phone conversation. The feedback from the questionnaire was generic so I am not sure I really understood what to interpret from that. |
|  |  | So far all the questions have been about one parent. What about the opinion of struggles of the second parent as these would be very different to mine. |

*Note.* N=851 parents who completed at least one question on the baseline acceptability questionnaire. At the end of the acceptability questionnaire, participants were asked to provide any feedback on their experiences of taking part and suggestions for improvements.

# Table S13: Summary of responses to closed questions on the 12-week acceptability questionnaire

| **Item** | **Response / mean (SD)** | **Intervention**  **N=434** | **Usual school practice**  **N=431** | **Total**  **N=865** |
| --- | --- | --- | --- | --- |
| I was happy to complete the questionnaires | Completely disagree | 1/342 (<1%) | 2/352 (<1%) | 3/694 (<1%) |
|  | Disagree | 1/342 (<1%) | 5/352 (1%) | 6/694 (<1%) |
|  | Neither agree nor disagree | 20/342 (6%) | 21/352 (6%) | 41/694 (6%) |
|  | Agree | 112/342 (33%) | 140/352 (40%) | 252/694 (36%) |
|  | Completely agree | 208/342 (61%) | 184/352 (52%) | 392/694 (56%) |
|  | Mean (SD), n | 4.54 (0.65), n=342 | 4.42 (0.72), n=352 | 4.48 (0.68), n=694 |
| Taking part in this study was helpful for me and/or my child | Completely disagree | 1/341 (<1%) | 5/354 (1%) | 6/695 (<1%) |
|  | Disagree | 2/341 (<1%) | 31/354 (9%) | 33/695 (5%) |
|  | Neither agree nor disagree | 14/341 (4%) | 133/354 (38%) | 147/695 (21%) |
|  | Agree | 83/341 (24%) | 118/354 (34%) | 201/695 (29%) |
|  | Completely agree | 241/341 (71%) | 67/354 (19%) | 308/695 (44%) |
|  | Mean (SD), n | 4.65 (0.62), n=341 | 3.60 (0.94), n=354 | 4.11 (0.96), n=695 |
| Taking part in this study was harmful for me and/or my child* | Completely disagree | 297/340 (87%) | 275/353 (78%) | 572/693 (83%) |
|  | Disagree | 30/340 (9%) | 55/353 (16%) | 85/693 (12%) |
|  | Neither agree nor disagree | 5/340 (1%) | 18/353 (5%) | 23/693 (3%) |
|  | Agree | 3/340 (<1%) | 5/353 (1%) | 8/693 (1%) |
|  | Completely agree | 5/340 (1%) | 0/353 (0%) | 5/693 (<1%) |
|  | Mean (SD), n | 1.20 (0.65), n=340 | 1.30 (0.63), n=353 | 1.25 (0.64), n=693 |
| I would recommend taking part in a similar study to other families and schools | Completely disagree | 1/339 (<1%) | 2/351 (<1%) | 3/690 (<1%) |
|  | Disagree | 0/339 (0%) | 9/351 (3%) | 9/690 (1%) |
|  | Neither agree nor disagree | 14 /339 (4%) | 95/351 (27%) | 109/690 (16%) |
|  | Agree | 102/339 (30%) | 145/351 (41%) | 247/690 (36%) |
|  | Completely agree | 222/339 (65%) | 100/351 (28%) | 322/690 (47%) |
|  | Mean (SD), n | 4.60 (0.60), n=339 | 3.95 (0.84), n=351 | 4.27 (0.80), n=690 |
| I was happy to work through the website | Completely disagree | 0/338 (0%) | - | - |
|  | Disagree | 0/338 (0%) | - | - |
|  | Neither agree nor disagree | 11/338 (3%) | - | - |
|  | Agree | 100/338 (30%) | - | - |
|  | Completely agree | 227/338 (67%) | - | - |
|  | Mean (SD), n | 4.64 (0.55), n=338 | - | - |
| The website was clear and easy to understand | Completely disagree | 0/338 (0%) | - | - |
|  | Disagree | 0/338 (0%) | - | - |
|  | Neither agree nor disagree | 10/338 (3%) | - | - |
|  | Agree | 122/338 (36%) | - | - |
|  | Completely agree | 206 (61%) | - | - |
|  | Mean (SD), n | 4.58 (0.55), n=338 | - | - |
| Working through the website was helpful for me and/or my child | Completely disagree | 1/336 (<1%) | - | - |
|  | Disagree | 1/336 (<1%) | - | - |
|  | Neither agree nor disagree | 15/336 (5%) | - | - |
|  | Agree | 111/336 (33%) | - | - |
|  | Completely agree | 208/336 (62%) | - | - |
|  | Mean (SD), n | 4.56 (0.63), n=336 | - | - |
| I was happy to have weekly calls with the MY-CATS Wellbeing Practitioner | Completely disagree | 0/339 (0%) | - | - |
|  | Disagree | 4/339 (1%) | - | - |
|  | Neither agree nor disagree | 14/339 (4%) | - | - |
|  | Agree | 87/339 (26%) | - | - |
|  | Completely agree | 234/339 (69%) | - | - |
|  | Mean (SD), n | 4.63 (0.62), n=339 | - | - |
| The support calls were helpful for me and/or my child | Completely disagree | 1/340 (<1%) | - | - |
|  | Disagree | 0/340 (0%) | - | - |
|  | Neither agree nor disagree | 12/340 (4%) | - | - |
|  | Agree | 78/340 (23%) | - | - |
|  | Completely agree | 249/340 (73%) | - | - |
|  | Mean (SD), n | 4.69 (0.57), n=340 | - | - |
| The online and telephone support was a good fit for me and my child | Completely disagree | 1/340 (<1%) | - | - |
|  | Disagree | 1/340 (<1%) | - | - |
|  | Neither agree nor disagree | 17/340 (5%) | - | - |
|  | Agree | 82/340 (24%) | - | - |
|  | Completely agree | 239/340 (70%) | - | - |
|  | Mean (SD), n | 4.64 (0.62), n=340 | - | - |
| The online and/or telephone support was harmful for me or my child in some way** | Completely disagree | 285/339 (84%) | - | - |
|  | Disagree | 21/339 (6%) | - | - |
|  | Neither agree nor disagree | 8/339 (2%) | - | - |
|  | Agree | 8/339 (2%) | - | - |
|  | Completely agree | 17/339 (5%) | - | - |
|  | Mean (SD), n | 1.38 (1.02), n=339 | - | - |
| I would recommend this support to other families | Completely disagree | 2/337 (<1%) | - | - |
|  | Disagree | 1/337 (<1%) | - | - |
|  | Neither agree nor disagree | 10/337 (3%) | - | - |
|  | Agree | 79/337 (23%) | - | - |
|  | Completely agree | 245/337 (73%) | - | - |
|  | Mean (SD), n | 4.67 (0.61), n=337 | - | - |

Data were not available for all participants. Data are n/N (%), unless otherwise stated

* 11 out 13 participants who agreed or completely agreed with the statement ‘Taking part in this study was harmful for me and/or my child’ also agreed or completely agreed with both of the statements ‘Taking part in the study was helpful for me/my child’ and ‘I would recommend taking part in a similar study to other families and schools’.

12 out 13 participants who agreed or completely agreed with the statement ‘Taking part in this study was harmful for me and/or my child’ did not detail any possible harms/adverse experiences

1 Usual school practice arm participant who agreed with the statement ‘Taking part in this study was harmful for me and/or my child’ provided detail of a possible harm/adverse experience: ‘some of the questions set off my feelings of anxiety or guilt’

**All 25 intervention arm participants who agreed or completely agreed with the statement ‘The online and/or telephone support was harmful for me or my child in some way’ also agreed or completely agreed with the statement ‘Working through the website was helpful for me and/or my child’

24 out 25 intervention participants who agreed or completely agreed with the statement ‘The online and/or telephone support was harmful for me or my child in some way’ also agreed or completely agreed with the statement ‘The support calls were helpful for me and/or my child’

None of the intervention participants who agreed or completely agreed with the statement ‘The online and/or telephone support was harmful for me or my child in some way’ detailed any possible harms/adverse experiences.

# Table S14: Summary participant feedback provided in free text responses on 12-week acceptability questionnaire (intervention arm, N=342)

| **Feedback category / subcategory** | **Frequency n/N (%)** | **Exemplar quotes** |
| --- | --- | --- |
| **Positive feedback about the intervention** | 167/342 (49%) |  |
| General positive feedback (including recommending it to others) | 36/342 (11%) | I would 1,000,000% recommend this is made available to all schools and all young people, whether you know your child has difficulties or not. |
|  |  | I had no idea what to expect, and was just curious. I was worried about the amount of time it would take. But everything was fine, and every minute spent was worth it. |
|  |  | The mix of online and telephone allowed the flexibility to fit this in around daily life - this made it much easier to complete the modules. |
|  |  | The Support was tremendous and very non-judgemental. Everything was done at our own pace and I couldn't ask for more. |
|  |  | I’ve been recommending the programme to anyone and everyone! Trying to crystallise the process into chat-able tips for friends. |
| Positive about the intervention strategies and/or the impact of the intervention | 155/342 (45%) | Working with my child and learning tools and techniques to help them has made our morning before school much easier. |
|  |  | Allowed us to talk more openly about worries. |
|  |  | It made me realise that it’s ok for my child to face and talk about their fears and to have an understanding to share some of my fears as well and it has made us even closer. |
|  |  | This study has given me skills to cope with my child's anxieties and has helped me to blame myself less about these. |
|  |  | Due to this not being my normal approach it took me some time to get my head round it and a long time working on the sections and plans. I'm glad I persevered as I have seen some changes in [my child]. |
|  |  | When starting the study some of the ideas and new things to try seem a little bit out there but since trying different approaches I found they work to better advantages than expected. |
|  |  | It has had a really positive impact on our family life, my child's confidence and helped her get over her fears really effectively. |
| Positive about the therapist support | 77/342 (23%) | [cwp name] was able to explain anything I wasn't clear on, was very reassuring and encouraging which made putting the teaching into practice a little easier. |
|  |  | When I found out that I would be having weekly phone calls as part of the study, I was concerned because I typically feel awkward on calls but my MY-CATS contact has been very welcoming and encouraging. |
|  |  | I sometimes found myself panicking because I hadn't worked through the module until a couple of days before the session (due to lack of time and other commitments) but our practitioner was very supportive and I never felt 'told off', which was great. |
|  |  | [cwp name] is very easy to talk to and always understood where I was coming from. |
|  |  | I was pleased when we found out that we were getting the weekly supportive calls as I found it motivated us to work through the modules and try new things more than if we had been left to "self teach". |
| **Reasons helpfulness was limited** | 8/342 (2%) |  |
| Child was not anxious | 1/342 (<1%) | My child wasn't anxious to begin with, so a lot of this seemed pointless. I was happy to participate because it doesn't hurt to learn techniques in case it comes up but I cant say he massively improved, because he doesn't have anxiety. |
| Current circumstances/timing | 6/342 (2%) | I would only feedback that the timing for us was not great. Starting at the start of the school holidays was a struggle. |
| Strategies | 1/342 (<1%) | My child did not react well to the techniques I was taught. |
| Negative experiences of the intervention | 3/342 (<1%) | At times it did make me upset, reflecting on my parenting skills and considering my own anxieties/stress levels and confidence issues. |
|  |  | I found it confrontational to begin, as holding up a mirror to my own challenges with anxiety - I felt responsible for the 'what have I done to my child'. As weeks went by, this self-critic passed. |
|  |  | My child did not react well to the techniques I was taught. |
| Suggested changes/improvements to the intervention | 19/342 (6%) | Have Teams links for appointments on the MY CATS platform. Mine often got lost in emails! |
|  |  | It would be better, I think, if the calls would be every two weeks. |
|  |  | I found it a bit tricky that after setting the goals, there was no option to change/ modify them. |
|  |  | The computer game was a bit tricky for my 6 year old and after a very excited initial reaction she did soon lose interest. |

*Note:* N=342 parents in the intervention arm who completed at least one question on the 12 week acceptability questionnaire. The questionnaire provided free-text boxes for participants to provide details about why taking part was/was not helpful/harmful, why the intervention was/was not harmful, and any further feedback about their experiences or suggestions for improvements. Participant responses were coded in multiple categories where appropriate.

# Table S15: Summary participant feedback provided in free text responses on 12-week acceptability questionnaire (Usual school practice arm, N=355)

| **Feedback category** | **Frequency n/N (%)** | **Exemplar quotes** |
| --- | --- | --- |
| Questionnaires provided an opportunity for reflection/improved understanding | 52/355 (15%) | I have become more aware of my child's feelings and understanding when she needs to have support. |
|  |  | It has helped me a little to focus in on what issues he's had. |
|  |  | It’s helpful to pause and reflect on my child and may parenting. |
|  |  | As we're not getting the phone calls/meetings that the other group get this study isn't that helpful to us. However the questionnaires make me think about the issues raised so that I'm more aware when dealing with [child name] (e.g. if she's talking about a stomach ache). |
|  |  | It gave me an idea about situations that can make my child more anxious and also helped me understand my own reactions to these situations. I believe I can now can handle such situations in a better way. |
| Positive about contributing to the research/receiving written support at the end of the study/from study team | 13/355 (4%) | Although we fell into the control group of the study knowing there is support if required and there will be support at the end of the study is reassuring. |
|  |  | The links given for advice have been helpful. |
|  |  | I am happy to answer the questions for this study knowing that it is contributing towards research. |
|  |  | It’s helpful, everyone I have spoken to is really nice, even the emails are worded nicely and put me at ease. |
| Helpfulness limited by being in the control arm | 51/355 (14%) | It has not helped me to learn any ways to help my son out. |
|  |  | Nothing has changed as not in the course group. |
|  |  | As we're in the control arm I haven't found it helpful. |
| Negative feedback about length/relevance/clarity of some questions or response formats | 18/355 (5%) | Some of the questions relate to you as the parent answering the question, but there is often the other parent to consider. |
|  |  | Some questions are tricky because it's difficult to think of specific examples if your child is not showing any issues at the time or recently. |
|  |  | It would be helpful to have an option in between 'Never' and 'Several days' for the questions about me. |
|  |  | The questions are rather vague and do not always allow context. |
| Change or lack of change for reasons unrelated to the trial | 3/355 (1%) | Where things may look like they've improved for my child (through questionnaires), they have, and that's through school, her confidence has grown massively over her time in reception with a lot of things. |
|  |  | I don't find myself or my child to be overly anxious except for everyday things you expect them to be worried about like the dark. |
| Discomfort/distress completing questionnaires | 2/355 (<1%) | Some of the questions set off my feelings of anxiety or guilt. |
|  |  | The parenting questions may make the parent worry their answers could make them be perceived as a bad parent. |
| Suggested changes to procedures | 6/355 (2%) | Liaise more with the schools to give them advice on what they could be doing. |
|  |  | Perhaps more advertising and incentives to get busy/apathetic/less literate parents on board? |
|  |  | Maybe the ability to see how you have replied previously. |

*Note:* N=355 parents in the Usual school practice arm who completed at least one question on the 12 week acceptability questionnaire. The questionnaire provided free-text boxes for participants to provide details about why taking part was/was not helpful/harmful, and any further feedback about their experiences or suggestions for improvements. Participant responses were coded in multiple categories where appropriate.

# Table S16: Summary of responses to closed questions on the 12-month acceptability questionnaire

| **Item** | **Response / mean (SD)** | **Intervention**  **N=434** | **Usual school practice**  **N=431** | **Total**  **N=865** |
| --- | --- | --- | --- | --- |
| I was happy to complete the questionnaires | Completely disagree | 1/300 (<1%) | 1/308 (<1%) | 2/608 (<1%) |
|  | Disagree | 3/300 (1%) | 1/308 (<1%) | 4/608 (<1%) |
|  | Neither agree nor disagree | 18/300 (6%) | 16/308 (5%) | 34/608 (6%) |
|  | Agree | 97/300 (32%) | 128/308 (42%) | 225/608 (37%) |
|  | Completely agree | 181/300 (60%) | 162/308 (53%) | 343/608 (56%) |
|  | Mean (SD), n | 4.51 (0.69), n=300 | 4.46 (0.64), n=308 | 4.49 (0.66), n=608 |
| I was happy to take part in the interview | Completely disagree | 1/301 (<1%) | 1/307 (<1%) | 2/608 (<1%) |
|  | Disagree | 1/301 (<1%) | 4/307 (1%) | 5/608 (<1%) |
|  | Neither agree nor disagree | 10/301 (3%) | 18/307 (6%) | 28/608 (5%) |
|  | Agree | 92/301 (31%) | 124/307 (40%) | 216/608 (36%) |
|  | Completely agree | 197/301 (65%) | 160/307 (52%) | 357/608 (59%) |
|  | Mean (SD), n | 4.60 (0.61), n=301 | 4.43 (0.69), n=307 | 4.51 (0.66), n=608 |
| Taking part in this study was helpful for me and/or my child | Completely disagree | 3/300 (1%) | 8/307 (3%) | 11/607 (2%) |
|  | Disagree | 3/300 (1%) | 26/307 (8%) | 29/607 (5%) |
|  | Neither agree nor disagree | 12/300 (4%) | 122/307 (40%) | 134/607 (22%) |
|  | Agree | 93/300 (31%) | 81/307 (26%) | 174/607 (29%) |
|  | Completely agree | 189/300 (63%) | 70/307 (23%) | 259/607 (43%) |
|  | Mean (SD), n | 4.54 (0.71), n=300 | 3.58 (1.01), n=307 | 4.06 (1.00), n=607 |
| Taking part in this study was harmful for me and/or my child* | Completely disagree | 266/298 (89%) | 239/304 (79%) | 505/602 (84%) |
|  | Disagree | 21/298 (7%) | 49/304 (16%) | 70/602 (12%) |
|  | Neither agree nor disagree | 2/298 (<1%) | 12/304 (4%) | 14/602 (2%) |
|  | Agree | 4/298 (1%) | 3/304 (1%) | 7/602 (1%) |
|  | Completely agree | 5/298 (2%) | 1/304 (<1%) | 6/602 (1%) |
|  | Mean (SD), n | 1.19 (0.67), n=298 | 1.28 (0.62), n=304 | 1.24 (0.65), n=602 |
| I would recommend taking part in a similar study to other families and schools | Completely disagree | 2/296 (<1%) | 2/304 (<1%) | 4/600 (<1%) |
|  | Disagree | 0/296 (0%) | 9/304 (3%) | 9/600 (2%) |
|  | Neither agree nor disagree | 17/296 (6%) | 78/304 (26%) | 95/600 (16%) |
|  | Agree | 84/296 (28%) | 124/304 (41%) | 208/600 (35%) |
|  | Completely agree | 193/296 (65%) | 91/304 (30%) | 284/600 (47%) |
|  | Mean (SD), n | 4.57 (0.67), n=296 | 3.96 (0.86), n=304 | 4.27 (0.83), n=600 |
| I was happy to work through the website | Completely disagree | 1/301 (<1%) | - | - |
|  | Disagree | 1/301 (<1%) | - | - |
|  | Neither agree nor disagree | 18/301 (6%) | - | - |
|  | Agree | 95/301 (32%) | - | - |
|  | Completely agree | 186/301 (62%) | - | - |
|  | Mean (SD), n | 4.54 (0.66), n=301 | - | - |
| The website was clear and easy to understand | Completely disagree | 1/299 (<1%) | - | - |
|  | Disagree | 1/299 (<1%) | - | - |
|  | Neither agree nor disagree | 14/299 (5%) | - | - |
|  | Agree | 114/299 (38%) | - | - |
|  | Completely agree | 169/299 (57%) | - | - |
|  | Mean (SD), n | 4.50 (0.64), n=299 | - | - |
| Working through the website was helpful for me and/or my child | Completely disagree | 1/301 (<1%) | - | - |
|  | Disagree | 3/301 (1%) | - | - |
|  | Neither agree nor disagree | 19/301 (6%) | - | - |
|  | Agree | 116/301 (39%) | - | - |
|  | Completely agree | 162/301 (54%) | - | - |
|  | Mean (SD), n | 4.45 (0.69), n=301 | - | - |
| I was happy to have weekly calls with the MY-CATS Wellbeing Practitioner | Completely disagree | 2/300 (<1%) | - | - |
|  | Disagree | 4/300 (1%) | - | - |
|  | Neither agree nor disagree | 11/300 (4%) | - | - |
|  | Agree | 93/300 (31%) | - | - |
|  | Completely agree | 190/300 (63%) | - | - |
|  | Mean (SD), n | 4.55 (0.70), n=300 | - | - |
| The support calls were helpful for me and/or my child | Completely disagree | 2/301 (<1%) | - | - |
|  | Disagree | 2/301 (<1%) | - | - |
|  | Neither agree nor disagree | 14/301 (5%) | - | - |
|  | Agree | 87/301 (29%)_ | - | - |
|  | Completely agree | 196/301 (65%) | - | - |
|  | Mean (SD), n | 4.57 (0.68), n=301 | - | - |
| The online and telephone support was a good fit for me and my child | Completely disagree | 3/299 (1%) | - | - |
|  | Disagree | 3/299 (1%) | - | - |
|  | Neither agree nor disagree | 20/299 (7%) | - | - |
|  | Agree | 93/299 (31%) | - | - |
|  | Completely agree | 180/299 (60%) | - | - |
|  | Mean (SD), n | 4.48 (0.75), n=299 | - | - |
| The online and/or telephone support was harmful for me or my child in some way** | Completely disagree | 243/299 (81%) | - | - |
|  | Disagree | 15/299 (5%) | - | - |
|  | Neither agree nor disagree | 9/299 (3%) | - | - |
|  | Agree | 15/299 (5%) | - | - |
|  | Completely agree | 17/299 (6%) | - | - |
|  | Mean (SD), n | 1.49 (1.14), n=299 | - | - |
| I would recommend this support to other families | Completely disagree | 3/300 (1%) | - | - |
|  | Disagree | 2/300 (<1%) | - | - |
|  | Neither agree nor disagree | 12/300 (4%) | - | - |
|  | Agree | 79/300 (26%) | - | - |
|  | Completely agree | 204/300 (68%) | - | - |
|  | Mean (SD), n | 4.60 (0.69), n=300 | - | - |

Data were not available for all participants. Data are n/N (%), unless otherwise stated

*11 out 13 participants who agreed or completely agreed with the statement ‘Taking part in this study was harmful for me and/or my child’ also agreed or completely agreed with both of the statements ‘Taking part in the study was helpful for me/my child’ and ‘I would recommend taking part in a similar study to other families and schools’.

12 out 13 participants who agreed or completely agreed with the statement ‘Taking part in this study was harmful for me and/or my child’ did not detail any possible harms/adverse experiences

1 Usual school practice participant who agreed or completely agreed with this statement reported an adverse experience/possible harm: ‘Some of the questions brought up difficult feelings. I think it's because of the introspection needed to complete the questionnaire, and the potentially upsetting subject’.

**30 out 32 who agreed or completely agreed with the statement ‘The online and/or telephone support was harmful for me or my child in some way’ also agreed or completely agreed with both of the statements ‘Working through the website was helpful for me and/or my child’ and ‘The support calls were helpful for me and/or my child’

31 out 32 participants who agreed or completed agreed with the statement The online and/or telephone support was harmful for me or my child in some way did not detail any adverse experiences or possible harms

1 intervention arm participant who agreed with the statement ‘The online and/or telephone support was harmful for me or my child in some way’ reported an adverse experience/possible harm: ‘very stressful at times, having to lock in a time and date when life happens’

# Table S17: Summary participant feedback provided in free text responses on 12-month acceptability questionnaire (intervention arm, N=301)

| **Feedback category / subcategory** | **Frequency n/N (%)** | **Exemplar quotes** |
| --- | --- | --- |
| **Positive comments about the intervention** | **177/301 (59%)** |  |
| General positive feedback (including recommending it to others) | 36/301 (12%) | I love how the team care about the children and families. I would whole heartedly say every parent should complete the MY-CATS programme, it is amazing for giving insight into your child. |
|  |  | I have found the process to be nicely paced and very useful. I would and have recommended it to parents I know. This is the only parenting advice I have officially received since having my children. Most advice and guidance you have to seek out through the internet and most is contradictory. |
|  |  | I am grateful for all I have learnt and been able to expand on my prior knowledge. |
| Positive about the intervention strategies and/or the impact of the intervention | 138/301 (46%) | I feel like it made me calmer and I began to think through how I addressed difficult situations. |
|  |  | They are easy tools to use and highly effective. My child has always been confident but the few little parts she needed assistance with we have tools to manage those. She can now use these in future life. She also uses the tools she has learnt and tries to tell other children about them. |
|  |  | I cannot explain the amazing impact this trial has had on my child. With all the techniques learnt throughout the trial, and implemented, the difference in my child in a year has been incredible. Whilst we still see the difficulties he experiences, he has overcome so many. |
|  |  | I've had a friend with a child going through anxiety and have been able to share this with them. They found it a great help and are doing better. |
|  |  | It opened my eyes to what my son was thinking & feeling, how it was often different to what I thought. Has made me much more empathic and able to speak about feelings and fears much more effectively. He is an enthusiastic, 'have a go' little boy now. |
|  |  | [Child name] has been more confident and we still say when she's nervous about something think of the mycats lady and she is usually more brave. |
|  |  | The study changed the way I approached my parenting when either of my children are anxious. I believe that it opened up a way of conversing with my children that hadn't happened previously. I feel better equipped to speak to them about situations that are causing them anxiety/nerves, and like I can help them manage those feelings better. |
| Positive about the therapist support | 33/301 (11%) | I particularly valued the 1:2:1 calls and don't think we would have done as well without that. |
|  |  | My practitioner, [cwp], was an absolute star. She was very patient with me as I had to change our appointments frequently to fit them around work and home commitments. I trusted her immediately and she really helped me and my child get the most out of the study. |
|  |  | The phone calls were eye opening, warm and supportive. I always gained something profound and useful from the conversations I had with [cwp]. |
| Helpfulness limited | 12/301 (4%) | I have not managed to keep using the strategies we learnt although I know I have access to them if I need them. |
|  |  | We have since had an ASD diagnosis. While we tried the techniques and they were interesting, they did not work for us. |
|  |  | I'm not sure much has changed. |
|  |  | Unfortunately we were not able to do as much with this trial as we wanted to. |
|  |  | I’m not a phone person. |
| Negative feedback about length/relevance/clarity of some questions or response formats | 7/301 (2%) | I did find it quite difficult to answer many of the questions in the surveys. |
|  |  | Too many questions. |
| Negative experiences of the intervention | 3/301 (<1%) | Very stressful at times, having to lock in a time and date when life happens. |
|  |  | I’m not a phone person. |
|  |  | I was so excited to take part and was very frustrated by what I felt was a very tick box approach. |
| Suggested changes/improvements to the intervention | 8/301 (3%) | I think it would have helped having my daughter involved in certain areas of the study. |
|  |  | I would have preferred for the calls to be further apart so I had time to put what we discussed into practice. |
|  |  | I think it might also be helpful to focus on parental anxieties and life experiences that may have led to higher levels of anxiety. |

*Note:* N=301 parents in the intervention arm who completed at least one question on the 12 month acceptability questionnaire. The questionnaire provided free-text boxes for participants to provide details about why taking part was/was not helpful/harmful, why the intervention was/was not harmful, and any further feedback about their experiences or suggestions for improvements. Participant responses were coded in multiple categories where appropriate.

# Table S18: Summary participant feedback provided in free text responses on 12-month acceptability questionnaire (Usual school practice arm, N=308)

| **Feedback category** | **Frequency n/N (%)** | **Exemplar quotes** |
| --- | --- | --- |
| Questionnaires provided an opportunity for reflection/improved understanding | 44/308 (14%) | Really enjoyed answering the questions. Gave me time to reflect on my son and how I think he has developed over the past year. |
|  |  | As part of the group who did not have the additional support in the beginning the trial still made me aware that the actions of parents can help or hinder an anxious child. |
|  |  | It helped to talk about my concerns and prompted me to seek help for my child. |
|  |  | Encouraged consideration and conversations regarding anxiety within our family. |
| Positive about contributing to the research/receiving written support at the end of the study/from study team | 10/308 (3 %) | I loved the way the researchers spoke to me, it was always with so much kindness, understanding and compassion and I thank them for that. |
|  |  | Any contact I have had with the research team has been very clear and professional. |
| Helpfulness limited by being in the control arm | 48/308 (16%) | I am happy to participate, for the good of the study, but feel I haven't gained any help with how to manage my child's fears and worries. |
|  |  | I don't feel that we have lost or gained anything by taking part. |
|  |  | As a part of the control group, I did not receive any support. |
| Negative feedback about length/relevance/clarity of some questions or response formats | 13/308 (4%) | The questionnaires can be a little repetitive and time consuming. |
|  |  | A tick box for questions not relevant such as siblings etc. also a box to put notes in as sometimes it depends on situation. |
| Discomfort/distress completing questionnaires | 2/308 (<1%) | Has made me question if I am the cause of her anxieties |
|  |  | Some of the questions brought up difficult feelings… and the potentially upsetting subjects. |

*Note:* N=308 parents in the Usual school practice arm who completed at least one question on the 12 month acceptability questionnaire. The questionnaire provided free-text boxes for participants to provide details about why taking part was/was not helpful/harmful, and any further feedback about their experiences or suggestions for improvements. Participant responses were coded in multiple categories where appropriate.

# Table S19: Reported Adverse Events

| **Who event relates to** | **Nature of event** | **Date of onset** | **Did the event relate to the intervention or trial procedures?** | **Outcome** | **Trial arm** |
| --- | --- | --- | --- | --- | --- |
| Parent (participant) | Distress when learned family was allocated to control (usual school practice) group | 22.2.2021 | Yes – trial procedures | Resolved – continued with study | Usual school practice |
| Child and parent (participant)  Child and parent (participant) | Data breach:  Email related to setting up an account for the OSI intervention intended for one parent was sent in error to second parent. Both parents were receiving the OSI intervention.  Email included parent and child first names. | 17.1.2022 | Yes – trial procedures | Resolved – continued with study | Intervention |
